# A network perspective on metabolic inconsistency

**DOI:** 10.1186/1752-0509-6-41

**Published:** 2012-05-14

**Authors:** Nikolaus Sonnenschein, José Felipe Golib Dzib, Annick Lesne, Sebastian Eilebrecht, Sheerazed Boulkroun, Maria-Christina Zennaro, Arndt Benecke, Marc-Thorsten Hütt

**Affiliations:** 1 School of Engineering and Science, Jacobs University Bremen, Campus Ring 1, 28759 Bremen, Germany; 2, Institut des Hautes Études Scientifiques — Centre National de la Recherche Scientifique, Bures-sur-Yvette, France; 3 LPTMC CNRS UMR 7600, Université Pierre et Marie Curie-Paris 6, 4 place Jussieu75252 Paris Cedex 05, France; 4 Vaccine Research Institute, INSERM U955, Institut Mondor de Recherche Biomédicale, Université Paris-Est Créteil, Créteil, France; 5 INSERM, U970, Paris Cardiovascular Research Center, Paris, France; 6, University Paris Descartes, Paris, France

## Abstract

**Background:**

Integrating gene expression profiles and metabolic pathways under different experimental conditions is essential for understanding the coherence of these two layers of cellular organization. The network character of metabolic systems can be instrumental in developing concepts of agreement between expression data and pathways. A network-driven interpretation of gene expression data has the potential of suggesting novel classifiers for pathological cellular states and of contributing to a general theoretical understanding of gene regulation.

**Results:**

Here, we analyze the coherence of gene expression patterns and a reconstruction of human metabolism, using consistency scores obtained from network and constraint-based analysis methods. We find a surprisingly strong correlation between the two measures, demonstrating that a substantial part of inconsistencies between metabolic processes and gene expression can be understood from a network perspective alone. Prompted by this finding, we investigate the topological context of the individual biochemical reactions responsible for the observed inconsistencies. On this basis, we are able to separate the differential contributions that bear physiological information about the system, from the unspecific contributions that unravel gaps in the metabolic reconstruction. We demonstrate the biological potential of our network-driven approach by analyzing transcriptome profiles of aldosterone producing adenomas that have been obtained from a cohort of Primary Aldosteronism patients. We unravel systematics in the data that could not have been resolved by conventional microarray data analysis. In particular, we discover two distinct metabolic states in the adenoma expression patterns.

**Conclusions:**

The methodology presented here can help understand metabolic inconsistencies from a network perspective. It thus serves as a mediator between the topology of metabolic systems and their dynamical function. Finally, we demonstrate how physiologically relevant insights into the structure and dynamics of metabolic networks can be obtained using this novel approach.

## Background

Genomic knowledge allows compiling an inventory of an organism’s enzymes and thus the subsequent reconstruction [1] and simulation of its metabolic system [2] using constraint-based modeling (CBM) techniques [3]. Compensating the lack of detailed information on the systems parameters, e.g., enzyme kinetics, gene regulation etc., CBM has proven to be a valuable tool for genome-scale system analysis. For example, flux balance analysis (FBA) [4] has been used to predict with high accuracy the lethality of gene deletions in unicellular organisms by taking only the metabolic system’s stoichiometry, the assumption of optimal growth (implicit gene regulation), and a specified growth medium into account (see e.g. [5], for a study involving *Escherichia coli* or [6], for a study involving *Saccharomyces cerevisiae*).

Duarte et al. [7], published a genome-scale representation of human metabolism based on genomic, bibliographic, and biochemical information. In contrast to metabolic representations of unicellular organisms, the following caveats play a role in the modeling of multicellular reconstructions, in general, and in particular for the human system [8]: (i) it is difficult to define environmental conditions for a multicellular system, (ii) usually not enough information is available about the cell-type specificity of human metabolic pathways, and (iii) cellular objectives, a prerequisite for flux balance analysis, are hard to define and validate. The precision of CBM predictions increase with the availability and accuracy of constraints, as they aid narrowing down the potential solution space to the biologically meaningful system states. Thus, integrating experimental data can help overcome the previously mentioned limitations.

In the present work, we will integrate human transcriptome data from a cohort of healthy controls and aldosterone producing adenomas (APA) of adrenal glands from primary aldosteronism (PAL) patients [9] with the metabolic reconstruction *Human Recon 1*[7]. Primary aldosteronism is a common form of hypertension with hypokalemia and suppressed renin-angiotensin system caused by autonomous aldosterone production. This data, among other data sets covered in the supporting information, will serve us to demonstrate how the metabolic contextualization dramatically increases the resolution of our perception of the data.

### Approach

**Figure 1:**
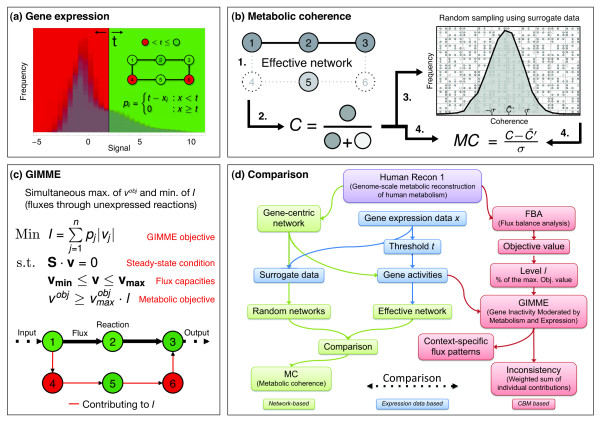
**A schematic figure explaining the metabolic coherence (*MC*), inconsistency *I, *and methodological approach behind the comparison of *MC *and *I. *(a)** A threshold *t* is applied onto the data *x*, depicted here as an overlay histogram of the raw log-signal intensities of the adenoma data, in order to obtain a binary gene/reaction (nodes) presence (green) and absence (red) pattern *p*. **(b)** Effective gene subnetworks are constructed from the present genes in *p* and the overall static gene-network representation of human metabolism (step 1). The ratio (coherence *C*) of connected genes (gray nodes) to isolated genes (light gray nodes) is determined (step 2). Sampling the overall static network with randomly generated presence patterns provides a distribution of null hypothesis coherence values *C’* (step 3). The coherence *C* is transformed into the z-score *MC* (metabolic coherence) using *C’* (step 4). **(c)** GIMME (Gene Inactivity Moderated by Metabolism and Expression) computes suitable flux distributions by simultaneously asserting flux through a specified objective (*v*^*obj*^; in fact a certain level *l* of the theoretically achievable maximum $\left(v_{\text{max}}^{\text{obj}}\right)$ determined by FBA) and minimizing flux through absent reactions. **(d)** The flow chart depicts the necessary steps in setting up a comparative analysis of *MC* and *I*.

Different approaches to the incorporation of experimental data into CBM have been proposed [8,10-13] (see Supporting Information Additional file 1: Text S1 for a summary). The GIMME (Gene Inactivity Moderated by Metabolism and Expression) algorithm, proposed by Becker and Palsson [14], maintains flux through a proposed metabolic objective (similar to FBA) by simultaneously punishing flux through unexpressed reactions. A threshold on the transcriptome data is used for expression classification purposes (see Figure 1a and c). The sum of fluxes through unexpressed reactions is termed inconsistency (*I*) and is minimized during the GIMME optimization. Thus, the inconsistency *I* gives, on the one hand, an estimate of the quality of the computed flux distribution, and measures, on the other hand, the coherence of the objective and the experimental data (see Materials and Methods and Supporting Information Additional file 1: Text S1). We chose GIMME over other methods for our analysis as (i) the threshold parameter used for determining gene presence-absence patterns is of particular interest to our study, and (ii) the inconsistency measure suits our approach of quantifying the discrepancy of the measured transcript levels to a given cellular objective, e.g., aldosterone or ATP production.

We will compare the inconsistency *I* to the metabolic coherence (*MC*) introduced previously [15], which is a purely topological quantity that measures the fragmentation of effective metabolic gene networks (see Figure 1b). The coherence of metabolic gene network topology and expression patterns is quantified as follows (see also Materials and Methods): in order to extract effective subnetworks, we map genes with expression values above threshold directly onto a metabolic gene network of human metabolism. Then, we compute the ratio of connected nodes and overall nodes in the effective subnetwork. This ratio is then converted into a z-score, by using a random distribution of expression changes as a null model (effectively choosing the same amount of affected nodes). This z-score is our *metabolic coherence* (*MC*), which measures the amount of network coherence between gene expression profiles and metabolic pathways.

The comparison between these two indices is interesting, as they highlight different properties of the network dynamics. The inconsistency index *I*, on the one hand, measures the level of disagreement between expression data and anticipated network dynamics. The *MC* index, on the other hand, measures the amount of coordinated (connected) expressed reaction structure, which can only be observed after contextualization of the expression data.

Figure 1d shows a flow diagram that describes the structure and necessary steps of our comparative analysis. Based on this quantitative comparison, we will conduct a topological characterization of the individual contributions to *I* and show that valuable information can be extracted from them.

## Results

### Inconsistency and metabolic coherence uncover two types of metabolic behavior

**Figure 2:**
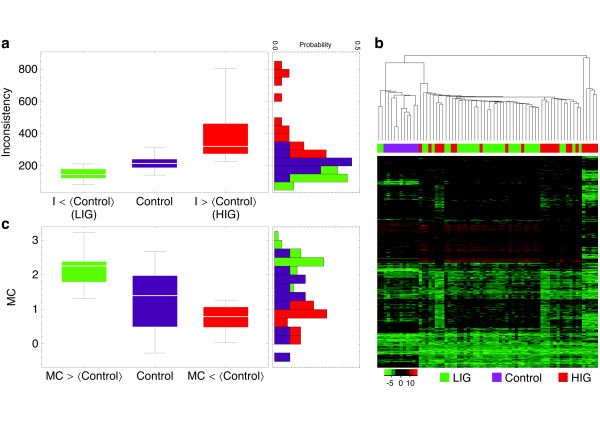
**Two types of metabolic behaviors in adenoma tumors.** Distributions of **(a)** inconsistency (*I*) and **(c)** metabolic coherence (*MC*) scores for the adenoma and control samples. **(b)** A hierarchical cluster-analysis of the data does not reveal a clear separation of the low (LIG) and high (HIG) inconsistency groups.

Using the GIMME approach, can physiological insights be obtained from the adenoma transcriptome data? Figure 2a shows the distribution of inconsistency values for the control and the adenoma transcriptome profiles. Maximal aldosterone production was used as the cellular objective function *v*^*obj*^ and a minimal medium composition containing glucose and glycerol, as well as a collection of amino acids and fatty acids, was implemented using the appropriate boundaries on the their respective exchange reactions (for further details see Materials and Methods and Supporting Information Additional file 1: Text S1). The histogram in Figure 2a uncovers a bimodal distribution of adenoma inconsistency values: it consists of a group of adenomas exhibiting lower(higher) inconsistencies than the average 〈*I*〉 of the control group. We term them high and low inconsistency group respectively (HIG and LIG). It is important to note that these two categories (HIG and LIG) could not have been uncovered by conventional cluster analysis (see Figure 2b).

How does the purely topological metabolic coherence method compare to the previously applied GIMME approach? Measuring the metabolic coherence for the adenoma transcript data reveals a similar pattern (see Figure 2c). Although not quite as distinct as for the inconsistency measure (compare to Figure 2a), two groups of high and low coherence are visible, which leads us to the following comparison of inconsistency and metabolic coherence.

### Comparison of metabolic coherence and inconsistency

We have shown above, that GIMME, as well as the metabolic coherence, permit an interrogation of the transcriptome data in a metabolic context. Both provide biological meaningful insights, which could not have been obtained by classical means of microarray data analysis. But how does GIMME compare to the metabolic coherence in detail, which is a purely topological score that inquires far less parameters and assumptions?

**Figure 3:**
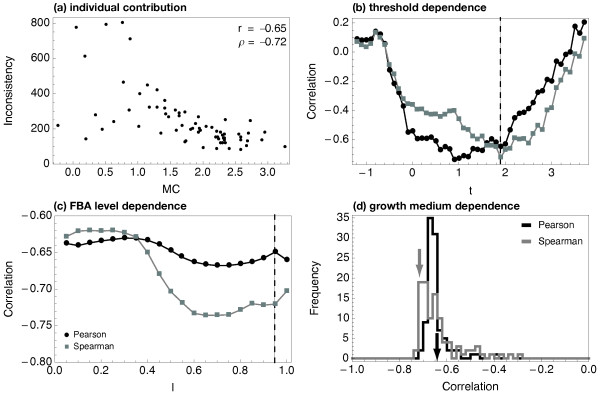
**Comparison of metabolic coherence and inconsistency measures for the adenoma data set. (a)** The aldosterone-production inconsistency values are plotted against the *MC* of 69 tumor and control data sets. A clear negative correlation is visible (Pearson’s product-moment correlation coefficient *r*=−0.65, with *p*≤7×10^−10^determined by one-tailed *t* statistic, and Spearman’s rank correlation coefficient *ρ*=−0.72; *t*=1.9; *l*=0.95). **(b)** Dependency of the correlation on the threshold parameter (*l*=0.95). **(c)** Medium dependency of the negative correlation strength. Both Spearman’s rank correlation coefficient as well as Pearson’s correlation where computed for the *MC* and the inconsistency for 100 random growth media (*t*=1.9; *l*=0.95). The dashed line in (b) indicates the threshold parameter used in (a). Arrows in (c) indicate the correlation values found in (a).

Figure 3a shows a scatterplot of the metabolic coherence and inconsistency values for all 69 expression profiles (58 adenomas + 11 controls), using the reference medium and the threshold *t*=1.9, revealing a strong anticorrelation between both measures (Pearson’s product correlation coefficient *r*=−0.65, with *p*≤7×10^−10^determined by one-tailed *t* statistic; Spearman’s rank correlation coefficient *ρ*=−0.72). The dependence of the correlation on the threshold parameter *t*, applied to the data in order to distinguish between expressed and not expressed metabolic genes, is checked in Figure 3b. The strongest negative correlation appears for parameter ranges that fit our statistical understanding of the raw signal distribution (see Figure 1a). No significant dependence on the level parameter *l* was found (see Figure 3c; *l* is used to enforce a certain flux through the stated objective *v*^*obj*^).

In Figure 3d the dependence of the correlation between inconsistency and the *MC* on the chosen growth medium is shown. The distribution of correlation coefficients is narrow (between −0.75 to −0.4 for *r* and −0.73 to −0.35 for *ρ*), regardless of which correlation measure is considered. This indicates that both inconsistency and *MC* and their correlation seem not be strongly dependent on the environmental conditions provided. Furthermore, the correlation values obtained for the reference medium (i.e. *r*=−0.64 and *ρ*=−0.68, see also above) seem to originate in the left tail of the distributions, suggesting a rather high correspondence with the *in vivo* situation.

We confirmed that the observed anticorrelation between both measures is not a feature of the adenoma data, but holds true for other transcriptome data as well [16] (see Figure S1 and S2 in Supporting Information Additional file 1: Text S1).

### Inconsistency contributions in central human metabolism

**Figure 4:**
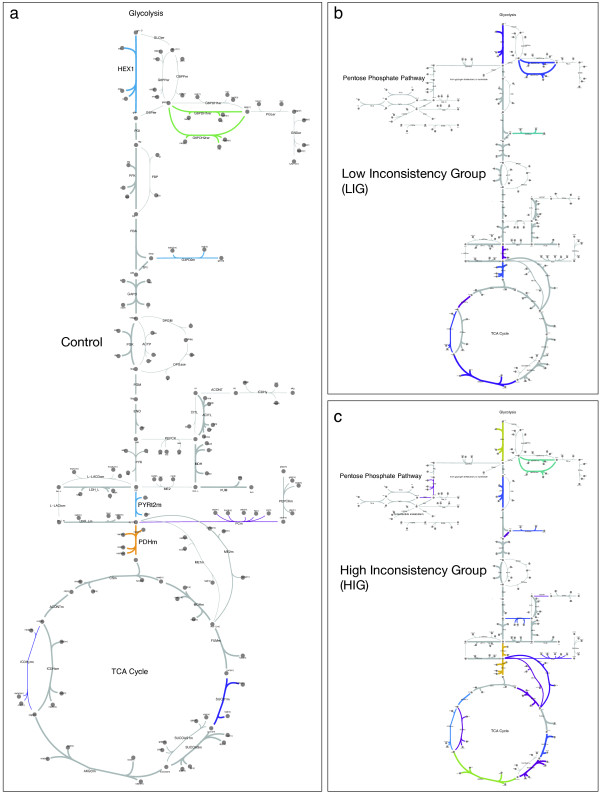
**Inconsistency contributions to carbohydrate metabolism.** The maps depict the usage patterns and inconsistency contributions for **(a)** the control and **(b)** the high and **(c)** low inconsistency groups (HIG and LIG). The thickness and color of a reaction edge correspond to the usage frequency and the contribution strength, respectively. The pathway maps have been obtained from the BIGG database [17].

Figure 4 displays inconsistency contributions and flux patterns for control, HIG and LIG on the carbohydrate metabolism pathways of *Homo sapiens*. Striking differences between the control group and the adenoma groups become visible in this overview, e.g., the pentose-phosphate pathway seems to be activated only in the HIG and LIG. Furthermore, the control pathway map lays out a rather consistent pattern of flux activities. Though, a few exceptions arise: pyruvate dehydrogenase (PDHm), the major entry point to the TCA cycle, and to lesser extent hexokinase (HEX1) and the pyruvate transport from cytosol to mitochondrium (PYRt2m), exhibit high contribution strengths. It is intriguing that the contribution strengths for these particular reactions is diminishingly small in the LIG case, which indicates an elevated energy metabolism for those adenomas. The HIG, on the other hand, shows a large number of reactions with elevated contribution strengths homogeneously distributed over the whole map, which indicates a significantly reduced energy metabolism.

### Individual contributions to the inconsistency

The correlation between metabolic coherence and inconsistency suggests a connection between both measures, and thus the possibility of interpreting the inconsistency values from the perspective of network topology. In order to investigate this point, we will decompose the inconsistency value into a vector of individual contributions, i.e., reactions that have been reinserted during the optimization procedure in order to achieve the targeted flux-level of the objective function. We further define the contribution strength of a reaction as the number of contributions it makes to the inconsistencies of a data set divided by the size of the respective data set.

**Figure 5:**
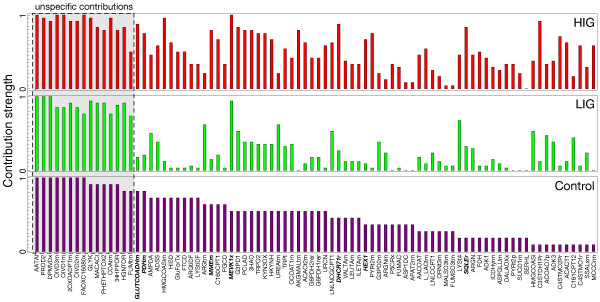
**Inconsistency contributions from adenoma tumor samples showing lower (LIG) and higher (HIG) inconsistencies (top and middle panel) in comparison to the control group (bottom panel).** The gray box highlights the group of unspecific reaction contributions. These contributions are covered together with a selection differentially contributing (bold reaction labels) in Table 1 and Supporting Additional file 1: Text S1 Table S2. Only a subset of all contributing reactions is shown due to space limitations (the complete diagram is available in Additional file 1: Text S1 Figure S3).

Comparing the contribution strengths of reactions in central metabolism reveals interesting changes in the physiology of LIG and HIG (see Figure 4). But how do the contribution strengths vary between control, LIG and HIG on a global metabolic scale? The contribution strengths for a subset of all contributing reactions is shown in Figure 5 (see Additional file 1: Text S1 Figure S3 for the complete set of contributions). The contributing reactions have been sorted according to their contribution strengths in the control group and at equal strength by the overall contributions. Having already seen a few examples for differentially contributing reactions in the carbohydrate pathways, it becomes evident that there are many more to discover.

On the other hand, a group of reactions with very high contribution strengths seems to contribute non-specifically and independently from the gene expression data. In the following, we want to elaborate on this set of reactions, and will use certain categories and topological markers to characterize them.

The following circumstances can lead to non-specific contributions to the inconsistency vector: 

1. A reaction is *expressedin vivo* but the measured gene expression intensity falls below the threshold *t* under most or all experimental conditions (*just below threshold*). This is a consequence of the rigid application of a universal threshold. Topologically, these contributions often disrupt a chain of otherwise expressed reactions (*chain disruptor*).

2. A reaction is *expressedin vivo* but, e.g., wrong GPR associations, missing isozymes, wrong gene annotations, erroneous data etc., make it invisible for the analysis. Again, these artifacts are often characterized by an interrupted chain of expressed reactions (*chain disruptor*).

3. The reaction is *not expressed*, but it has to be utilized by GIMME due to the following reasons: 

(a) The stated objective function does not reflect the situation present in the cell. Defining the objective functions as the output of the system, these reactions contributions should often lie close to it (*close to output layer*).

(b) The chosen media composition does not reflect the *in vivo* environment in which the experimental data has been obtained. The preliminary FBA step in GIMME is naive about the *in vivo* medium composition and uses everything provided and suitable for the maximization of the objective function. As GIMME enforces a certain achievement of the objective flux predicted by FBA, many of the transport reactions used by FBA will also be used by GIMME. Topologically, these reaction contributions are characterized by lying close to the provided medium components (*close to input layer*).

(c) Too many missing gene-protein-reaction associations (GPR), either due to non-enzymatic reaction steps or knowledge gaps, before and after the contributing reaction can lead to wrongly activated paths, as missing GPR information is not punished by GIMME (*invisible path*).

(d) Alternative *expressed* routes to the objective function are available *in vivo*, but are not covered by the metabolic reconstruction. This leads to reaction contributions that are characterized by producing essential precursors for the objective function, and thus constitute bottlenecks in the system (*bottleneck*).

Table 1 lists topological and biological classifications for a selection of the 11 unspecific contributions as well as 8 selected differentially contributing reactions (see Figure 5; the full listing is provided in Supporting Additional file 1: Text S1 Table S1). The topological characterization from the enumeration above have also been applied to the specific contributions.

Our network-based approach is capable of identifying similar patterns as the more sophisticated methods based on flux-balance analysis. In this way, our approach can facilitate an understanding of metabolic inconsistencies from a network perspective. It thus serves as a mediator between the topology of metabolic systems and their dynamical function. In the following we will discuss two representative sets of contributions to the inconsistency in greater detail.

**Table 1:** **Classification of contributions to the inconsistency vector (*bottleneck; *BN, *invisible path; *IP, *chain disruptor; ***CD, *close to input layer; ***CIL, *close to output layer; *COL, SIaa; Additional file**2**, SIcarb; Additional file**3**, SIlip; Additional file**4**, SIvit; Additional file**5**)**

**Contributor**	**Category**	**Topological class**	**Biological interpretation**	**Reference**
AATAi^**^	unspecific	BN; IP	*2-Aminoadipate transaminase*;one out of two 2-oxoadipate producing reactions; missing GPR assoc.	
			in all precursors.	SIaa, B1
PROD2^*^	unspecific	CD; CIL	*Proline dehydrogenase*; participates in a cycle that converts nadh to fadh2 (Figure 6); not expressed (Figure S5b).	SIaa, D3
DPMVDx	unspecific	CD	*Diphosphomevalonate decarboxylase*; essential step in the cholesterol biosynthesis pathway;	
			not expressed; wrong or missing GPR assoc. (Figure S5c).	SIlip, B5
GLYK	unspecific	CIL	*Glycerol kinase*; not expressed in control and LIG, indicating that glycerol (provided in the *in silico* medium)	
			might not be available as a *in vivo* medium component; slightly elevated expression levels in LIG (see Figure S5i).	SIlip, E5
PHETHPTOX2	unspecific	CIL	*Phenylalanine 4-monooxygenase*; converts phenylalanine (provided in the *in silico* medium) into tyrosine	
			(not provided in the *in silico* medium); the high unspecific contribution strength indicates that tyrosine might be	
			available as an *in vivo* medium component.	SIaa, A5
34HPPOR^***^	unspecific	CD; CIL	*4-Hydroxyphenylpyruvate dioxygenase*; involed in tryosine to fumarate and acetoacetate conversion;	
			not expressed (see Figure S5m).	SIaa, B5
FUMtm	unspecific	—	*Fumarate transport (cytosol/mitochondria)*; expression just below threshold (see Figure S5o).	no map
GLUTCOADHm^**^	specific	CD	*Glutaryl-CoA dehydrogenase*; involved in the 2-oxoadipate pathway (see Figure S5); elevated expression levels in LIG (see Figure S6a).	SIaa, A2
PDHm	specific	BN	*Pyruvate dehydrogenase*; entry point to the TCA cycle; elevated expression levels in LIG (see Figure S6c).	SIcarb, C3
MMEm	specific	CD	*Methylmalonyl-CoA epimerase*; involved in isoleucine degradation; slightly elevated expression levels in LIG (see Figure S6d).	SIaa, D1–E1
MEVK1x	specific	CD	*Mevalonate kinase*; an essential step in cholesterol biosynthesis; decreased expression levels in LIG and HIG (see Figure S6e).	SIlip, B5
G6PDH(1,2)rer	specific	–	*Glucose-6-phosphate dehydrogenase*; slightly elevated expression levels in LIG (see Figure S6f).	SIcarb, C4–D4
DHCR71r	specific	CD; COL	*7-Dehydrocholesterol reductase*; involved in cholesterol biosynthesis; slightly elevated expression levels in LIG (see Figure S6g).	SIlip, A4
HEX1	specific	CD; CIL	*Hexokinase*; first step in glycolysis; slightly elevated expression levels in LIG (see Figure S6h).	SIcarb, C4–C5
SQLEr	specific	CD	*Squalene epoxidase*; decreased expression levels in LIG and HIG (see Figure S6i).	SIlip, A5

**Figure 6:**
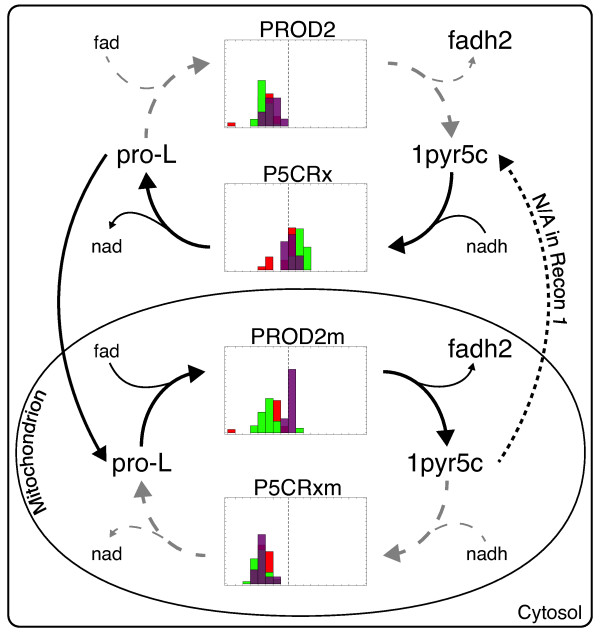
***Δ***^**1**^**-pyrroline-5-carboxylate-proline cycle.** The NADH to FADH2 interconverting cycle composed of *pyrroline-5-carboxylate reductase* and *pyrroline-5-carboxylate reductase* is depicted together with the distributions of expression values for the cytosolic (PROD2 and P5CRx) and mitochondrial (PROD2m and P5CRxm) versions of *pyrroline-5-carboxylate reductase* and *proline dehydrogenase*. The control is depicted in purple, the LIG and HIG in green and red, respectively, and the dashed lines indicate the threshold used for the GIMME computations (see Figure 7 for a detailed legend).

*Proline cycle issue; see Figure 6.

**Figure 7:**
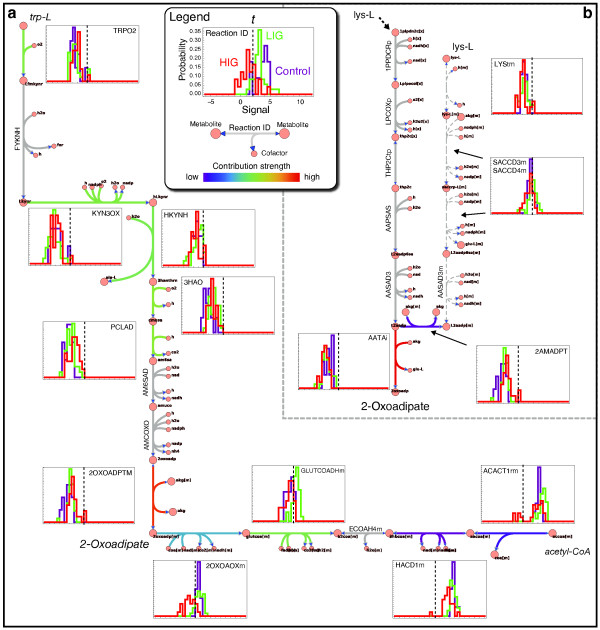
**(a) 2-Oxoadipate production pathway starting from lysine and involving the unspecific contributor AATAi (see Table**1**and Supporting Additional file**1**: Text S1 Table S2).** Dashed lines indicate the threshold used for the GIMME computations. **(b)** 2-Oxoadipate production pathway starting from tryptophan and involving the unspecific contributor 2OXOADPTm (see Table 1 and Supporting Additional file 1: Text S1 Table S2) among other more specific contributions. Dashed lines indicate the threshold used for the GIMME computations.

**2-Oxoadipate issue; see Figure 7.

***Tyrosine path to fumarate and acetoacetate issue; see Figure S4 and Text S1.

### *Δ*^1^-pyrroline-5-carboxylate-proline cycle

Proline dehydrogenase (PROD2) emerges as one of the major unspecific inconsistency contributors (see Table 1). Together with *Δ*^1^-pyrroline-5-carboxylate reductase (P5CRx), it is involved in a cycle that interconverts NADH into FADH2 (see Figure 6), a necessary redox factor for the biosynthesis of cholesterol biosynthesis, the ultimate precursor for all steroid pathways and concomitantly aldosterone production. The cycle involves synthesis and degradation of L-proline, where *Δ*^1^-pyrroline-5-carboxylate (1pyr5c) acts as precursor as well as degradative product. In the cytosol, P5CRx seems to be expressed in most of the samples, whereas expression levels for PROD2 fall all below the GIMME threshold. It is intriguing that the gene expression profiles are almost reversed in the mitochondrion, where PROD2m (a mitochondrial version of PROD2) is expressed (at least in the control) and P5CRxm (a mitochondrial version of P5CRx) is not expressed. It is known that the distinct reaction steps of the *Δ*^1^-pyrroline-5-carboxylate-proline cycle are localized over different subcellular locations [18]: (i) the dehydrogenation of proline to 1pyr5c takes place in the mitochondrion (i.e. by PROD2m), (ii) 1pyr5c emerges from the mitochondrion and (iii) is converted back to proline in the cytosol, (iv) which is then transported back into the mitochondrion, closing the cycle. In fact, these are also the steps suggested by the observed expression patterns. So why does GIMME predict the cycle to take place exclusively in the cytosol, although PROD2 expression is clearly absent in all samples? Checking the model revealed missing mitochondrial transporters for 1pyr5c (a transporter for proline is available), prohibiting its correct physiological operation and suggesting necessary amendments to the human model. Furthermore, the reduced expression of PROD2m in LIG and HIG constitutes an interesting deviation to metabolic signature of the control group.

### 2-Oxoadipate pathways

2-Oxoadipate (2oxoadp) is one of the precursors for acetyl-CoA (see Figure 7a), which is heavily utilized in cholesterol biosynthesis. Only two paths lead to 2-oxoadipate, i.e., L-tryptophan (Figure 7a) and L-lysine degradation (Figure 7b). The many missing GPR associations (Invisible pathway) on the path leading from lysine to 2-oxoadipate (Figure 7b), surely promote the usage of this specific pathway versus the alternative pathway leading from tryptophan to 2-oxoadipate (Figure 7a), explaining the high contribution strength of AATAi (see Figure 5 and Table 1). The expression data suggests the absence of both catabolic pathways. However, it is intriguing to see that all subsequent steps from 2-oxoadipate to acetyl-CoA seem to be expressed in the control and LIG (Figure 7a), implying that 2-oxiadipate might still be metabolized in the samples. Further investigations in this direction might be promising, especially in the light of the elevated expression levels (LIG vs. control) found for glutaryl-CoA dehydrogenase (GLUTCOADHm) and acetyl-CoA C-acetyltransferase (ACACT1rm).

## Discussion

There is an ongoing interest in the generic [19] and network-based [20] properties of metabolic systems, though discussions of metabolic systems from a network perspective have frequently been criticized and are prone to artifacts, when one attempts to biologically interpret the observed topological properties. Table 1 on the other hand shows, how a topological perspective can help guide the biological interpretation of experimental data and constraint-based analysis results. Classifying metabolic inconsistencies from a topological perspective allowed us to think of such inconsistencies in terms of bottlenecks, paths and branching ratios, etc. As an extension to this work we would like to formalize our approach in the future.

Comparing the contribution strengths of individual reactions among the different sample categories (control, LIG, HIG) revealed unspecific contributions to the inconsistency, as well as a group of reactions that differentially contribute in a specific fashion. We constructed a methodological framework for the topological classification of the inconsistency contributions. Therefore, topological markers were developed for the characterization of both, specific and unspecific contributions, thus enabling a thorough understanding of the context-specific flux-activity results. It turned out, that on the one hand, the specific contributions cast light on an unforeseen diversity of alterations in the physiology of adrenal gland adenomas and, on the other hand, the unspecific contributions provide entry points for the iterative refinement of the metabolic reconstruction.

## Conclusion

We have presented a sequence of three results on the network-mediated correspondence between gene expression patterns and metabolic systems: (1) We have shown the general agreement between GIMME [14] and a purely topological method from [15], both of them capable to detect distinct physiological behaviors in the adrenal gland tumors. (2) We have extended the GIMME approach by moving from the inconsistency score to the inconsistency vector that contains the various contributions to the metabolic inconsistency. (3) We have been able to formulate biological hypotheses for these vector components based on comparison with network topology.

## Methods

An extended “Methods” section is provided in the Supporting Information.

### Model of human metabolism

All flux balance simulations were conducted using the metabolic reconstruction Recon 1, a genome-scale compartmentalized representation of human metabolism [7], which is available in SBML [21] format via the BIGG database [17].

### Gene expression data

Aldosterone producing adenomas were obtained through the COMETE network from patients who had undergone surgery for lateralized PAL at the Hôpital Européen Georges Pompidou between 2002 and 2006. Methods for screening and criteria for diagnosing PAL were in accordance with institutional guidelines and have been described recently [22]. The clinical and biological characteristics of the patients are resumed in Boulkroun et al. [23]. Here, logarithmized transcript levels from 58 adenomas and 11 control tissue samples were mapped onto the GPR (gene-protein-reaction) associations included in the Human Recon 1 model. Therefore, it was necessary to replace logical AND and OR by *min* and *max* functions, respectively, following the protocol described in [14]. The eleven control normal adrenals (CA) were obtained from enlarged nephrectomies (kindly provided by the department of Pathology of the University Hospital of Rouen, Hôpital Tenon as described previously [9]). The EBER2 gene expression has been published in [16].

### Context-specific flux balance analysis

Context-specific flux balance analysis of human expression data was conducted using the GIMME algorithm as described in [14] and in the introduction to this work. ATP-production was implemented as a cellular objective by introducing an artificial reaction that consumes cytosolic ATP. The aldosterone objective was implemented as the maximization of flux through aldosterone synthase (model ID: P45011B21m). The pathway to aldosterone was initially blocked in the metabolic reconstruction. Further analysis revealed 4-Methylpentanal as a dead-end metabolite inhibiting steady-state flux to the aldosterone synthase reaction. The introduction of an artificial drain for 4-Methylpentanal restored the functionality of the whole pathway. Furthermore, the same conservative approach was chosen regarding missing GPR: reactions without GPR associations were assumed to be expressed, i.e., having expression values above *t*. The aldosterone objective and the parameters *t*=2 and *l*=0.8 were used throughout the study, if not stated otherwise.

### Growth media

The growth medium was defined as in [24] (see Table S1 in Supporting Information Additional file 1: Text S1). It contains both glucose and glycerol as carbon sources, the amino acids L-arginine, L-histidine, L-isoleucine, L-leucine, L-lysine, L-Methionine, L-phenylalanine, L-threonine and L-tryptophane, as well as the fatty acids palmitic and linoleic acid. Aerobic conditions were assumed by leaving oxygen consumption unconstrained. Random media conditions were constructed by picking randomly between 4% (approx. the number of enabled exchange reactions in the reference medium) to 100% of all available exchange reactions in the model and assigning random upper and lower boundaries in the intervals $\left[-20,0\right]$ and $\left[0,20\right]$ to them. Oxygen, protons, sulfate, phosphate, water were assumed to be always available. In case of random media sampling, inconsistency values *I* have been normalized by the objective function’s flux in order to make them comparable.

### Metabolic coherence

The metabolic coherence (*MC*) was computed as described in [15]. Active genes *g* (obtained after thresholding) are mapped onto a gene network (representing metabolism) *G* to obtain an effective subgraph *G*^*sub*^ that consists of nodes *g*^*sub*^=*g*∩*V*(*G*). Repeatedly and randomly choosing |*g*^*sub*^| nodes from *G* provides us with a distribution of *N* random subgraphs *G*^*rnd*^. Let *C*(*G*) be a function that extracts the set of connected nodes from a network. Let

(1) $$\mu =\frac{1}{N}\overset{N}{\underset{i=1}{\sum }}\frac{\left|C(G_{i}^{\text{rnd}})\right|}{\left|g^{\text{sub}}\right|}$$

denote the mean of the ratio of connected nodes to overall nodes in the population of random networks and

(2) $$\sigma =\sqrt{\frac{1}{N}\overset{N}{\underset{i=1}{\sum }}{\left(\frac{\left|C(G_{i}^{\text{rnd}})\right|}{\left|g^{\text{sub}}\right|}-\mu \right)}^{2}}$$

its standard deviation. The z-score that we term metabolic coherence can then be described as

(3) $$\mathrm{MC}=\frac{\frac{\left|C(G_{\text{sub}})\right|}{\left|g_{\text{sub}}\right|}-\mu }{\sigma }.$$

Before constructing the gene network out of the bipartite representation, overly abundant currency metabolites, e.g., ATP, H_2_O, NADH etc., have been excluded by removal of 4% of the highest connected compounds from each compartment in the network [25]. The presented results, e.g., the strong anti-correlation between *MC* and inconsistency, are not particularly sensitive to this parameter (see Additional file 1: Text S1 Supplementary Figure S7).

## Competing interests

The authors declare that they have no competing interests.

## Authors’ contributions

NS, JFGD, AL, SB, MCZ, AB, and MTH conceived and designed the experiments. NS, JFGD, and SB performed the experiments. NS and JFGD analyzed the data. AL, SE, MCZ, and AB contributed materials and analysis tools. NS, AB, and MTH wrote the manuscript. All authors read and approved the final manuscript.

## Supplementary Material

Additional file 1 Text S1. Supporting information, including Supplemental Figures S1–7 and Tables S1–2 [8-14,16,21-24,26-28]. Click here for file

Additional file 2 **Pathway map SIaa.** Amino acid biosynthesis pathways. The map depicts the usage patterns and inconsistency contributions for the overall contributions (page i), control (page ii), LIG (page iii), and HIG (page iv). The thickness and color of a reaction edge corresponds to the usage frequency and the contribution strength, respectively. The pathway maps have been obtained from the BIGG database [17]. Click here for file

Additional file 3 **Pathway map SIcarb.** Central metabolism and carbohydrate pathways (see also Additional file 2. Click here for file

Additional file 4 **Pathway map SIlip.** Lipid metabolism pathways (see also Additional file 2. Click here for file

Additional file 5 **Pathway map SIvit.** Vitamins and cofactor pathways (see also Additional file 2. Click here for file
