# Supplementary material for: A network perspective on metabolic inconsistency
Source: BMC Syst Biol. 2012 May 14;6:41. doi: 10.1186/1752-0509-6-41 (PMC3579709; doi:10.1186/1752-0509-6-41)
Supplement: Additional file 1 — Text S1. Supporting information, including Supplemental Figures S1–7 and Tables S1–2 [8-14,16,21-24,26-28]. [file 1752-0509-6-41-S1.pdf]

Text S1: Supporting Information for "A Network Perspective on Metabolic Inconsistency"

Gene expression data.

Aldosterone producing adenomas were obtained through the COMETE network from patients who had undergone surgery for lateralized PAL at the Hôpital Européen Georges Pompidou between 2002 and 2006. Methods for screening and criteria for diagnosing PAL were in accordance with institutional guidelines and have been described recently [11]. The clinical and biological characteristics of the patients are resumed in Boulkroun et al. [4]. Eleven control normal adrenals (CA) were obtained from enlarged nephrectomies (kindly provided by the department of Pathology of the University Hospital of Rouen, Hôpital Tenon as described previously [5]). Total RNA isolation from the tissues as well as procedures for labeling and hybridization to Applied Biosystems AB1700 Human Genome Survey Arrays has been described previously [2]. This transcriptome profiling technique is considered to be of particularly high sensitivity [12], and raw data were quality controlled using recently described procedures [6]. Here, logarithmized transcript levels from 58 adenomas and 11 control tissue samples were mapped onto the GPR (gene-protein-reaction) associations included in the Human Recon 1 model. Therefore, it was necessary to replace logical AND and OR by *min* and *max* functions, respectively, following the protocol described in [3]. The EBER2 gene expression has been published in [8].

Context-specific flux balance analysis.

The optimization problem which is solved by GIMME can be formulated in the following way:

$$\begin{aligned} \text{Minimize} \quad & I = \sum_{j=1}^{n} p_j |v_j| \\ \text{subject to} \quad & \mathbf{S} \cdot \mathbf{v} = 0 \\ & \mathbf{v_{min}} \leq \mathbf{v} \leq \mathbf{v_{max}} \\ & v^{obj} \geq v^{obj}_{max} \cdot l. \end{aligned} \tag{1}$$

The inconsistency score I is, technically speaking, the sum of all fluxes going through unexpressed reactions weighted by the respective experimental data p_j. Furthermore, n is the number of reactions/fluxes v, $\mathbf{S}$ represents the stoichiometry of the system as a matrix, and v^{obj}_{max} is the maximal flux through the

proposed objective reaction v^{obj} (v^{obj}_{max} is determined in a previous step by standard FBA without taking the experimental data into account). The condition $v^{obj} \geq v^{obj}_{max} \cdot l$ forces the system to operate at or above some level l chosen from the interval $(0, 1]$. The norm $|v_j|$ can be omitted by using exclusively irreversible reactions. This can be achieved by replacing reversible reactions with pairs of irreversible reactions.

The weighting vector p_j is constructed in the following way:

$$p_j = \begin{cases} t - x_j & \text{if} \quad x_j < t \\ 0 & \text{if} \quad x_j \geq t \end{cases} , \tag{2}$$

where t is a threshold applied to the gene expression data x that classifies reactions as either expressed ($x_j \geq t$) or not expressed ($x_j < t$) using the gene expression data $\mathbf{x}$. Fluxes through expressed reactions are thus not minimized in equation (1). Conversely, the usage (reinsertion) of fluxes through unexpressed reactions are weighted by the distance of the expression level from the threshold $t - x_j$ in equation (1).

Context-specific flux balance analysis of human expression data was conducted using the GIMME algorithm as described in [3] and in the introduction to this work. ATP-production was implemented as a cellular objective by introducing an artificial reaction that consumes cytosolic ATP. The aldosterone objective was implemented as the maximization of flux through aldosterone synthase (model ID: P45011B21m). The pathway to aldosterone was initially blocked in the metabolic reconstruction. Further analysis revealed 4-Methylpentanal as a dead-end metabolite inhibiting steady-state flux to the aldosterone synthase reaction. The introduction of an artificial drain for 4-Methylpentanal restored the functionality of the whole pathway. Furthermore, the same conservative approach was chosen regarding missing GPR: reactions without GPR associations were assumed to be expressed, i.e., having expression values above t. The aldosterone objective and the parameters $t = 2$ and $l = 0.8$ were used throughout the study, if not stated otherwise.

Expression data integration into constraint-based modeling approaches

Different approaches have been proposed for the incorporation of experimental data into CBM:

Akesson et al. [1] exploit the fact that under steady state conditions the absence of gene expression coincides with the corresponding protein unavailability, and thus inactivity of the corresponding reactions.

Thus, the flux through an enzymatically catalyzed reaction is constrained to zero if the gene corresponding to the enzyme is not expressed in an experimental data set.

The GIMME (Gene Inactivity Moderated by Metabolism and Expression) algorithm proposed by Becker and Palsson [3] relaxes the rigid approach described by Akesson et al. [1] by reinserting unexpressed reactions back into the system if a proposed cellular objective is not achieved. The sum over these reinserted fluxes is termed inconsistency (I) and is minimized during the GIMME optimization. The inconsistency I gives, on the one hand, an estimate of the quality of the computed flux distribution, and measures, on the other hand, the coherence of the objective and the experimental data.

Shlomi et al. [14] proposed a mixed-integer optimization problem formulation that allows the computation of tissue-specific, steady-state flux-distributions that match experimental data at hand as close as possible. In particular, it is not necessary to formulate a cellular objective function. The model building algorithm (MBA) published by [10] goes even a step further by using multiple experimental and bibliomic data sources to prune a generic model into a full-fledged, tissue-specific model.

In contrast to the previously described approaches, the E-flux method developed by Colijn et al. [7] uses experimental data directly as boundaries in the linear programming formulation. It has been successfully utilized for a comprehensive study of *Mycobacterium tuberculosis* mycolic acid metabolism predicting accurately the effects of a series of anti-TB drugs [7].

All previously described approaches (with the exception of the E-flux method [7]) have in common that they rely on subjective and somewhat arbitrary thresholds for the classification of genes into discrete categories, e.g., *expressed* and *not expressed* Metabolic Adjustment by Differential Expression (MADE), as developed by Jensen and Papin [9], is the newest in the family of methods for integrating gene expression data with metabolic networks. It eliminates the need for arbitrary thresholding by taking the statistical significance of differentially expressed genes into account, rendering it suitable for time series expression profiles.

Anticorrelation of MC and I: different dataset and objective

It is noteworthy, that a qualitatively similar negative correlation between metabolic coherence and inconsistency was also found using a more generic objective function, i.e., ATP production S1. Furthermore, in order to investigate if the observed anticorrelation between both measures holds true for other data, especially where the inconsistency difference between patient and control data is not as pronounced as for

the adenoma data, we recapitulate the results from the adenoma data for a strikingly different dataset (see Figure S2) and objective (ATP production). This data were obtained by overexpression of the viral EBER2 RNA, as well as a mutant derivative thereof in a human cell culture system. Detailed information on this study can be found in [8]. We currently ignore the mechanism by which EBER2 RNAs control cellular metabolism, however, point out that these effects are strikingly correlated when comparing the GIMME and MC methods, and again would neither have been appreciable using other methodology, nor reflect an underlying structure in the data that could have been uncovered by other methodology.

Supporting information for Table 1 (main manuscript)

Pathway maps

Pathways maps, referenced in Table 1 (SIaa, SIcarb, SIlip, SIvit), are provided electronically due to size limitations. The maps depict the usage patterns and inconsistency contributions for the overall contributions (page i), control (page ii), LIG (page iii), and HIG (page iv). The thickness and color of a reaction edge corresponds to the usage frequency and the contribution strength, respectively. The pathway maps have been obtained from the BIGG database [13].

Path from tyrosine to fumarate and acetoacetate

Tyrosine, which is not provided in the *in silico* medium (see Table S1), and is build from phenylalanine under a strong contribution of PHETHPTOX2 (*phenylalanine 4-monooxygenase*), seems to be involved in another highly contributing pathway that leads from tyrosine to fumarate and acetoacetate (see Figure S4). The entry and output point, TYRTA (*tyrosine transaminase*) and FUMAC (*fumarylacetoacetase*), of this chain of reactions seem to be expressed, whereas the intermediate steps, 34HPPOR (*4-hydroxyphenylpyruvate dioxygenase*), HGNTOR (*homogentisate 1,2-dioxygenase*), and MACACI (*maleylacetoacetate isomerase*), are all unspecific contributors enlisted in Table 1 and Supporting Table S2.

Supporting Figures

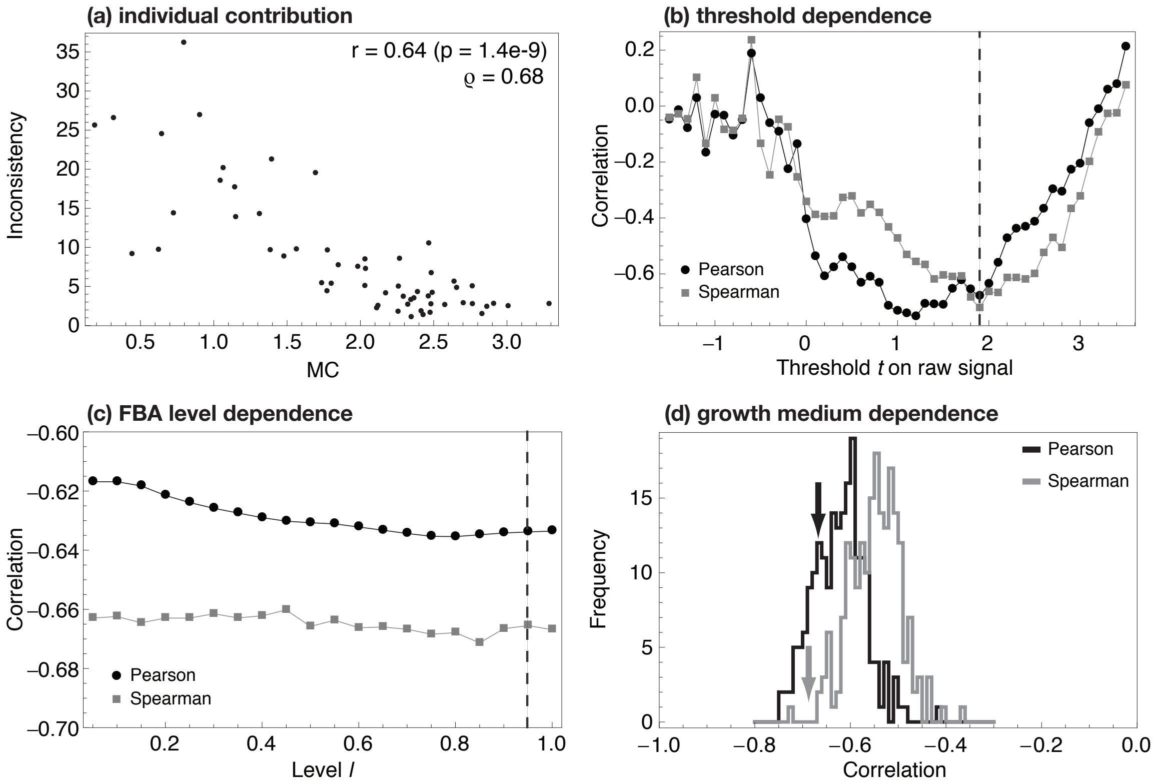

Supplementary Fig. S1. Comparison of metabolic coherence and inconsistency measures. (a) The ATP-production inconsistency values are plotted against the MC of 69 tumor and control data sets. A clear negative correlation is visible (Pearson's product-moment correlation coefficient $r = -0.64$, with $p \leq 1.4 \times 10^{-9}$ determined by one-tailed t statistic, and Spearman's rank correlation coefficient $\rho = -0.68$; $t = 1.9$; $l = 0.95$). (b) Dependency of the correlation on the threshold parameter ($l = 0.95$). (c) Dependency of the correlation on the level parameter ($t = 1.9$). (d) Medium dependency of negative correlation strength. Both Spearman's rank correlation coefficient as well as Pearson's correlation where computed for the MC and the inconsistency for 100 random growth media ($t = 2.$; $l = 0.95$). Dashed lines in (b) and (c) indicate the parameters that have been used in (a). Arrows in (d) indicate the correlation values found in (a).

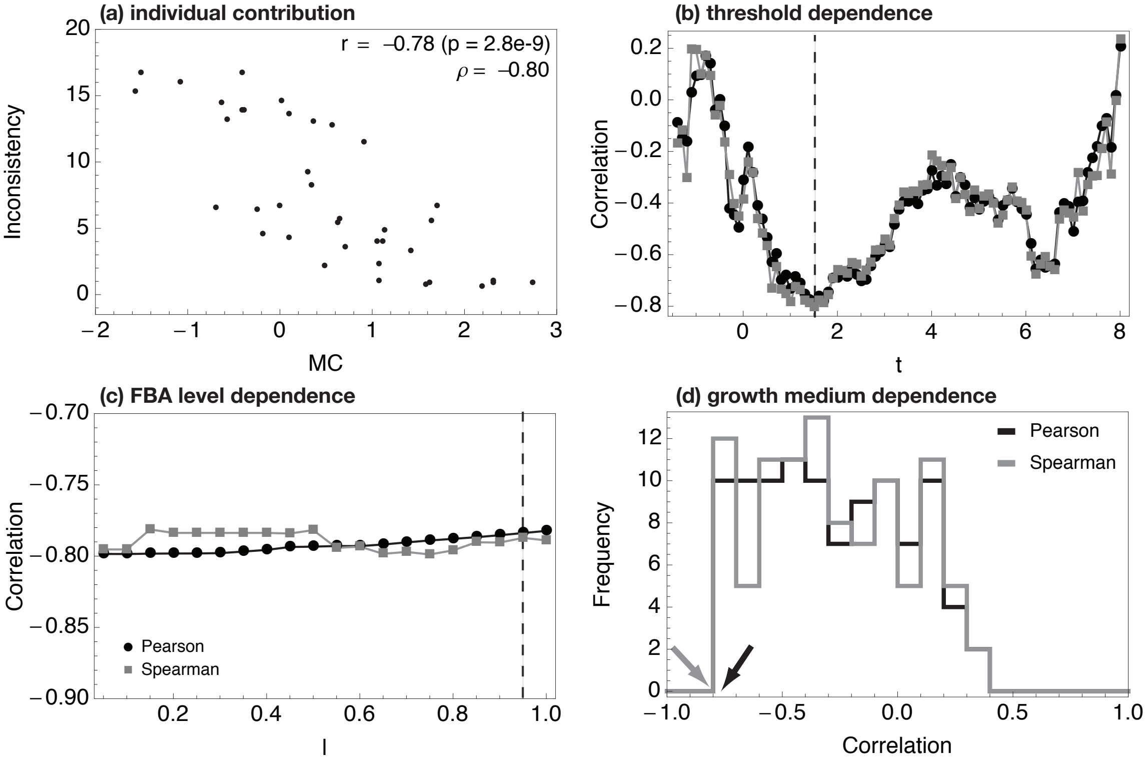

Supplementary Fig. S2. Comparison of metabolic coherence and inconsistency measures for the EBER2 data set. (a) The ATP-production inconsistency values are plotted against the MC of the different EBER2 transfection studies [8]. A clear negative correlation is visible (Pearson's product-moment correlation coefficient $r = -0.78$, with $p \leq 2.8 \times 10^{-9}$ determined by one-tailed t statistic, and Spearman's rank correlation coefficient $\rho = -0.80$; $t = 1.5$; $l = 0.95$). (b) Dependency of the correlation on the threshold parameter ($l = 0.95$). (c) Dependency of the correlation on the level parameter ($t = 1.5$). (d) Medium dependency of negative correlation strength. Both Spearman's rank correlation coefficient as well as Pearson's correlation where computed for the MC and the inconsistency for 100 random growth media ($t = 1.5$; $l = 0.95$). Dashed lines in (b) and (c) indicate the parameters that have been used in (a). Arrows in (d) indicate the correlation values found in (a).

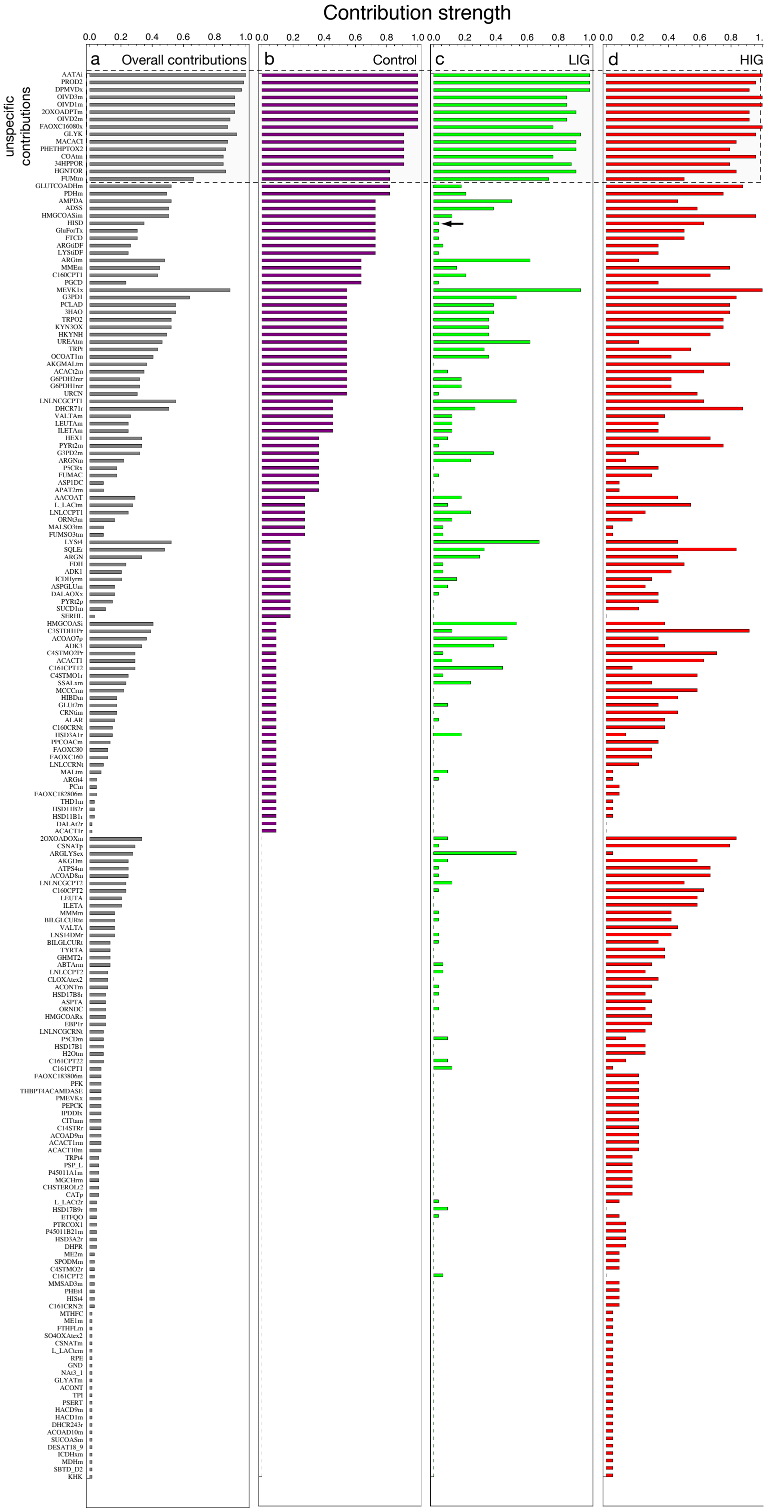

Supplementary Fig. S3. Inconsistency contributions. (a) Overall contributions to the inconsistency for all adenoma and control samples, (b) the control group, and the adenoma tumor samples showing (c) lower and (d) higher inconsistencies in comparison to the control group. The contributions have been normalized by the sample size, respectively. Only a subset of all contributing reactions is shown due to space limitations.

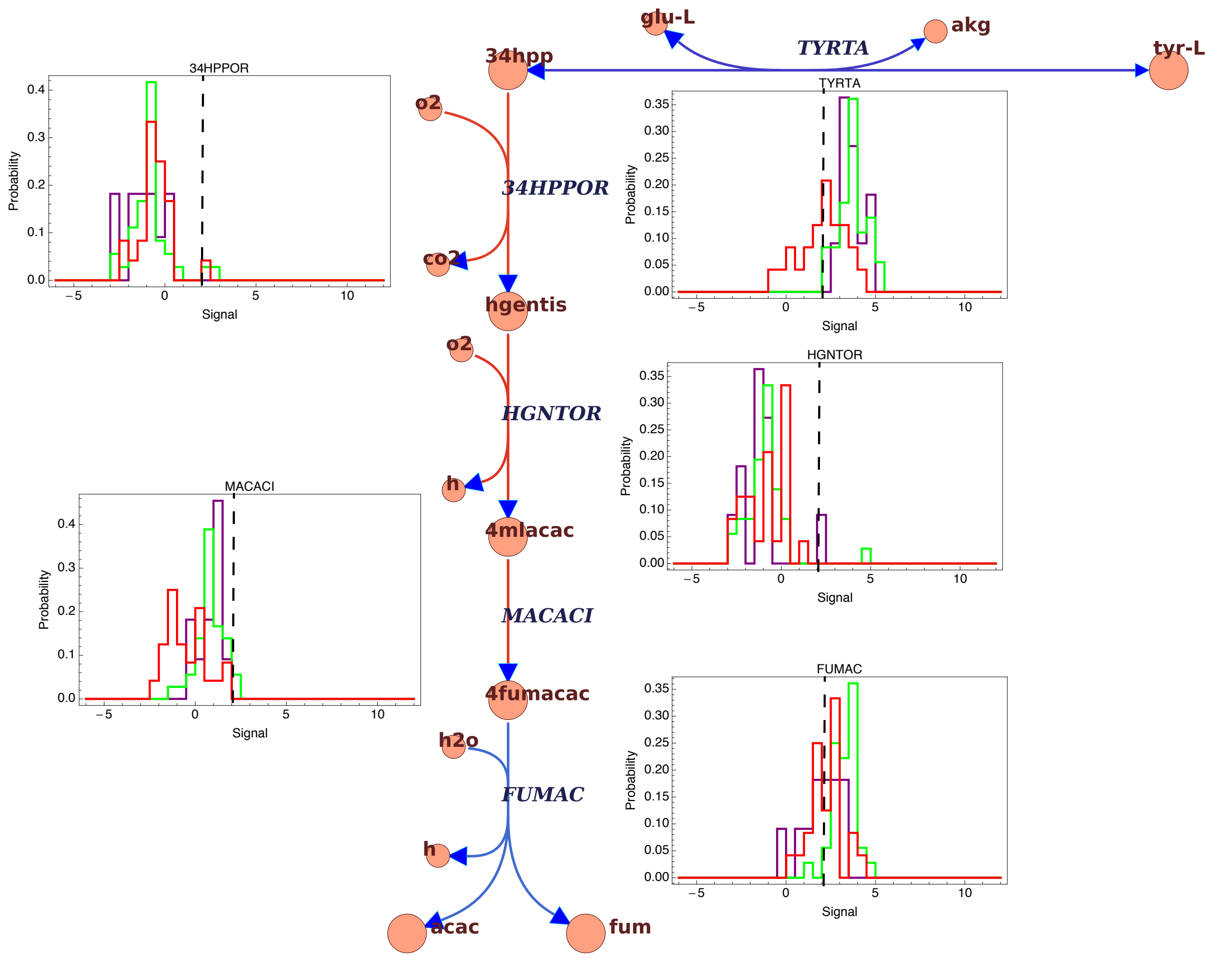

Supplementary Fig. S4. Pathway leading from tyrosine to fumarate and acetoacetate. Dashed lines indicate the threshold used for the GIMME computations.

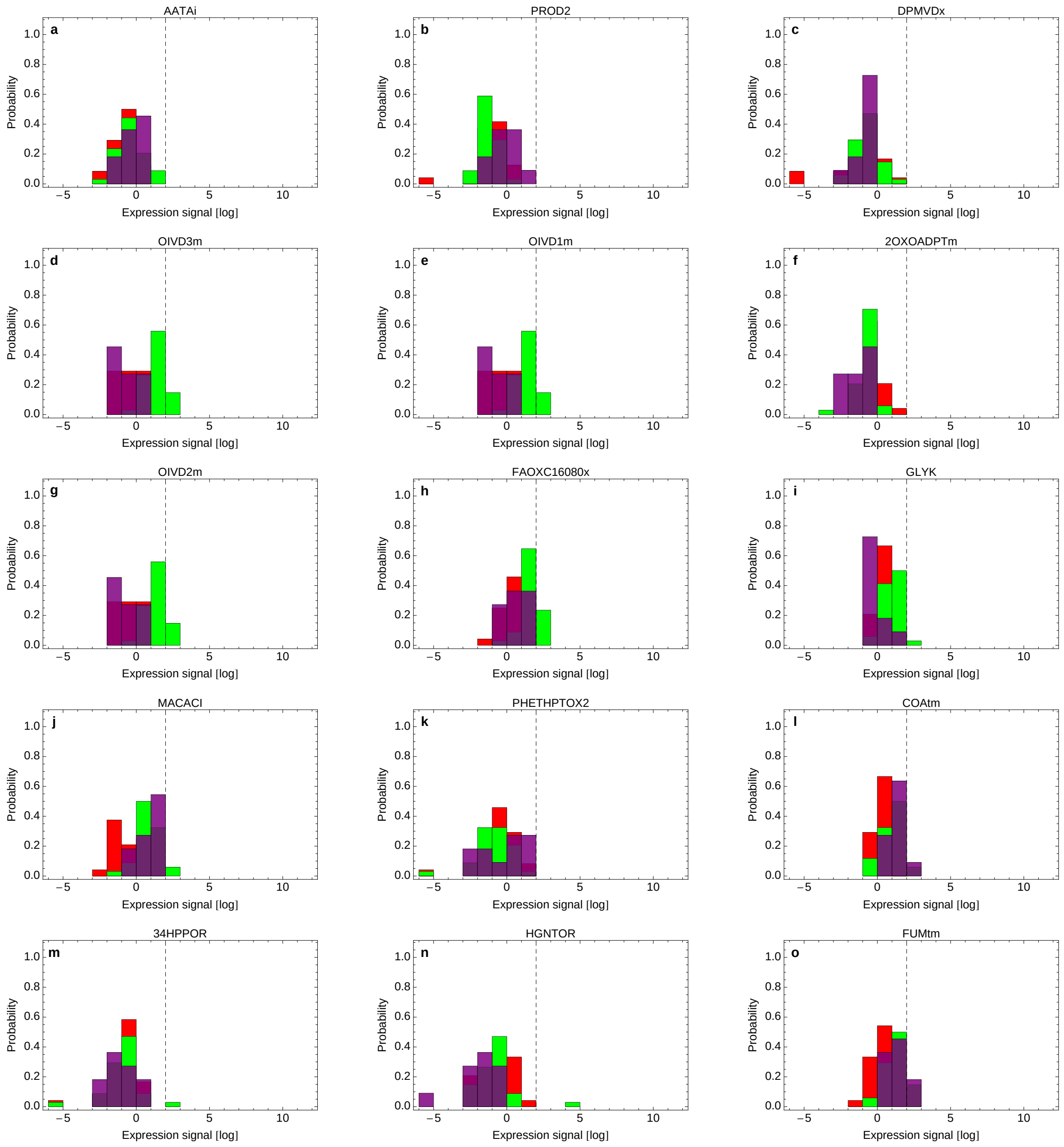

Supplementary Fig. S5. Distributions of reactions expression values of the unspecific contributions in Table 1 and Supporting Table S2. The control is depicted in purple, the LIG and HIG in green and red, respectively, and the dashed line indicates the threshold used for the GIMME computations.

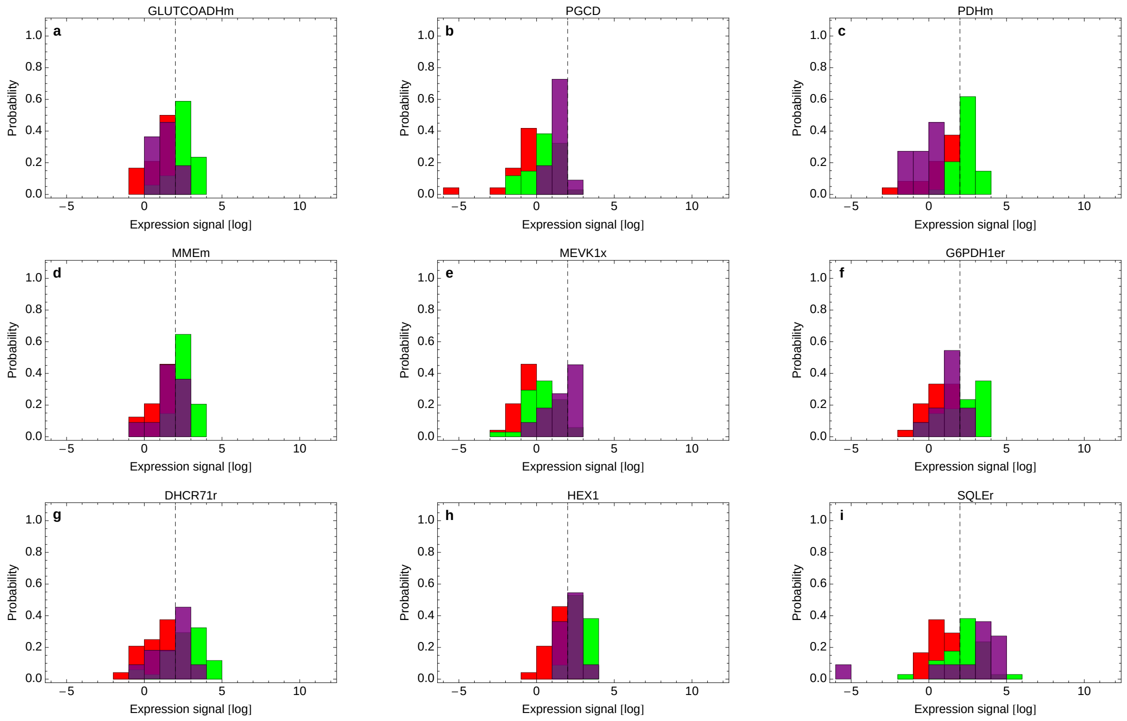

Supplementary Fig. S6. Distributions of reactions expression values of the specific contributions in Table 1 and Supporting Table S2. The control is depicted in purple, the LIG and HIG in green and red, respectively, and the dashed line indicates the threshold used for the GIMME computations.

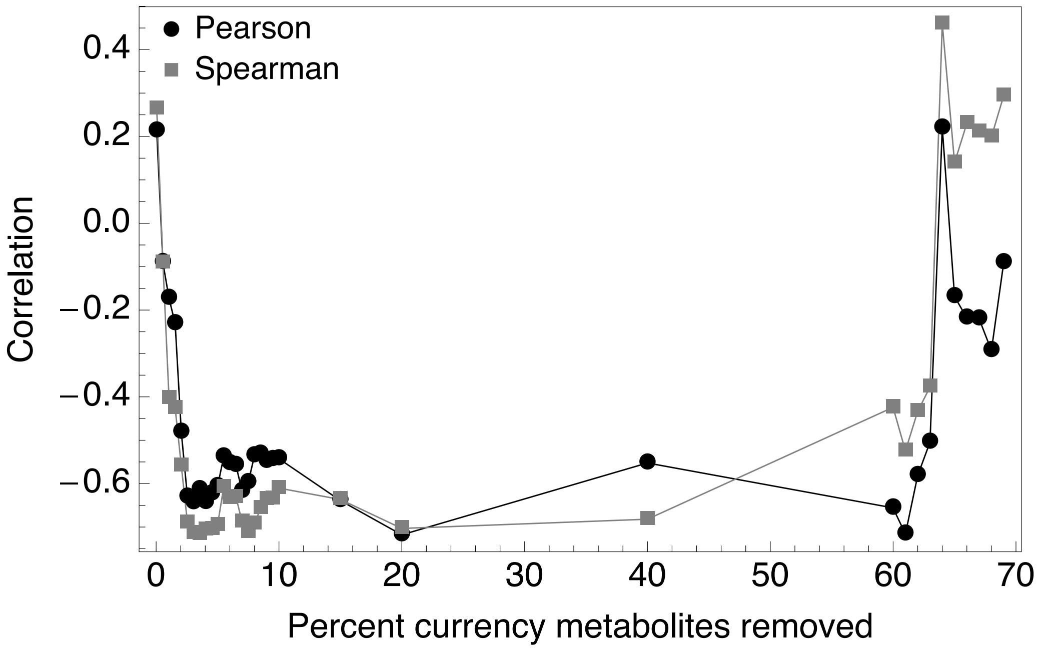

Supplementary Fig. S7. Currency metabolite removal. The negative correlation between MC and inconsistency (as shown in Figure 3a) is not particularly sensitive to the amount of currency metabolites removed from the metabolic network. In particular, the MC and inconsistency are uncorrelated for very low (0–2%; not enough ... connections between genes have been removed) and high percentages (64–70%; the pruned are too small to obtain a signal). from the network

Supplementary Table S1. Medium condition from [15]. Exchange reactions not mentioned in the table default to a lower bound (uptake) of 0 and an upper bound (secretion) of 10000. DM reactions not mentioned in the table default to a lower(upper) bound of 0(10000).

Reaction name	Lower bound	Upper bound
DM_13-cis-oretn(n)	0	0
DM_13-cis-retn(n)	0	0
DM_avite1(c)	0	0
DM_avite2(c)	0	0
DM_bvite(c)	0	0
DM_yvite(c)	0	0
EX_arg-L(e)	-1	10000
EX_fe2(e)	0	0
EX_glc(e)	-1	10000
EX_glyc(e)	-1	10000
EX_h(e)	-1	10000
EX_h2o(e)	-1	10000
EX_hdca(e)	-1	10000
EX_his-L(e)	-1	10000
EX_ile-L(e)	-1	10000
EX_leu-L(e)	-1	10000
EX_lnlc(e)	-1	10000
EX_lys-L(e)	-1	10000
EX_met-L(e)	-1	10000
EX_o2(e)	-10000	10000
EX_phe-L(e)	-1	10000
EX_pi(e)	-1	10000
EX_so4(e)	-1	10000
EX_thr-L(e)	-1	10000
EX_trp-L(e)	-1	10000
EX_val-L(e)	-1	10000

Supplementary Table S2. Classification of contributions to the inconsistency vector. Reactions are sorted according to their contribution strength and follow the same order as depicted in Figure S5. All unspecific and a few selected specific contributions are provided. Topological characterizations as well as biological interpretations are given. References point to positions on the electronically provided pathway maps (see Appendix).

Contributor	Category	Topological class	Biological interpretation	Reference
AATAi	unspecific	bottleneck; invisible path	*2-Aminoadipate transaminase*; one out of two 2-oxoadipate producing reactions; missing GPR assoc. in all precursors.	SIaa, B1
PROD2	unspecific	chain disruptor; close to input layer	*Proline dehydrogenase*; participates in a cycle that converts nadh to fadh2 (Figure 6 in the main manuscript and Appendix); not expressed (Figure S5b).	SIaa, D3
DPMVDx	unspecific	chain disruptor	*Diphosphomevalonate decarboxylase*; essential step in the cholesterol biosynthesis pathway; not expressed; wrong or missing GPR assoc.? (Figure S5c).	SIlip, B5
OIVD(1,2,3)m	unspecific	chain disruptor; close to input layer	*2-Oxoisovalerate dehydrogenase*; necessary for leucine(valine, isoleucine) processing; expression just below threshold (Figures S5d, e and g).	SIaa, D2–E2
2OXOADPTm	unspecific	bottleneck; invisible path	*2-Oxoadipate shuttle (cytosol/mitochondria)*; involved in the 2-oxoadipate producing pathway; missing GPR assoc. in precursor reactions.	SIaa, A2
GLYK	unspecific	close to input layer	*Glycerol kinase*; not expressed in control and LIG, indicating that glycerol (provided in the *in silico* medium) might not be available as a *in vivo* medium component; slightly elevated expression levels in LIG (see Figure S5i).	SIlip, E5
MACACI	unspecific	chain disruptor; close to input layer	*Maleylacetoacetate isomerase*; expression levels just below threshold (see Figure S5j).	SIaa, B5
PHETHPTOX2	unspecific	close to input layer	*Phenylalanine 4-monooxygenase*; converts phenylalanine (provided in the *in silico* medium) into tyrosine (not provided in the *in silico* medium); the high unspecific contribution strength indicates that tyrosine might be available as an *in vivo* medium component.	SIaa, A5
COAtm	unspecific	—	*CoA transporter (cytosol/mitochondria)*; expression just below threshold (see Figure S5i).	no map
34HPPOR	unspecific	chain disruptor; close to input layer	*4-Hydroxyphenylpyruvate dioxygenase*; involed in tryosine to fumarate and acetoacetate conversion; not expressed (see Figure S5m).	SIaa, B5
HGNTOR	unspecific	chain disruptor; close to input layer	*Homogentisate 1,2-dioxygenase*; involed in tryosine to fumarate and acetoacetate conversion; not expressed (see Figure S5n).	SIaa, B5
FUMtm	unspecific	—	*Fumarate transport (cytosol/mitochondria)*; expression just below threshold (see Figure S5o).	no map
GLUTCOADHm	specific	chain disruptor	*Glutaryl-CoA dehydrogenase*; involved in the 2-oxoadipate pathway (see Figure 7 in the main manuscript); elevated expression levels in LIG (see Figure S6a).	SIaa, A2
PGCD	specific	bottleneck	*Phosphoglycerate dehydrogenase*; expression is just below the threshold in the control, decreased expression levels in LIG an HIG (see Figure S6b).	SIaa, E5
PDHm	specific	bottleneck	*Pyruvate dehydrogenase*; entry point to the TCA cycle; elevated expression levels in LIG (see Figure S6c).	SIcarb, C3
MMEm	specific	chain disruptor	*Methylmalonyl-CoA epimerase*; involved in isoleucine degradation; slightly elevated expression levels in LIG (see Figure S6d).	SIaa, D1–E1
MEVK1x	specific	chain disruptor	*Mevalonate kinase*; an essential step in cholesterol biosynthesis; decreased expression levels in LIG and HIG (see Figure S6e).	SIlip, B5
G6PDH(1,2)rer	specific	topo	*Glucose-6-phosphate dehydrogenase*; slightly elevated expression levels in LIG (see Figure S6f).	SIcarb, C4–D4
DHCR71r	specific	chain disruptor; close to output	*7-Dehydrocholesterol reductase*; involved in cholesterol biosynthesis; slightly elevated expression levels in LIG (see Figure S6g).	SIlip, A4
HEX1	specific	chain disruptor; close to input layer	*Hexokinase*; first step in glycolysis; slightly elevated expression levels in LIG (see Figure S6h).	SIcarb, C4–C5
SQLEr	specific	chain disruptor	*Squalene epoxidase*; decreased expression levels in LIG and HIG (see Figure S6i).	SIlip, A5

2-Oxoadipate issue; see Figure 7 Tyrosine path to fumarate and acetoacetate issue; see Figure S4

References

1. Mats Akesson, Jochen Förster, and Jens Nielsen. Integration of gene expression data into genome-scale metabolic models. *Metab Eng*, 6(4):285–93, 2004.

2. J Barik, S Parnaudeau, A Lampin-Saint-Amaux, B P Guiard, J F Golib-Dzib, O Bocquet, A Bailly, A Benecke, and F Tronche. Glucocorticoid receptors in dopaminoceptive neurons, key for cocaine, are dispensable for molecular and behavioral morphine responses. *Biol. Psychiatry.*, 68:231–9, 2010.

3. Scott A Becker and Bernhard Ø Palsson. Context-specific metabolic networks are consistent with experiments. *PLoS Comput Biol*, 4(5):e1000082, 2008.

4. S Boulkroun, B Samson-Couterie, J F Golib-Dzib, L Amar, P F Plouin, M Sibony, H Lefebvre, E Louiset, X Jeunemaitre, T Meatchi, A Benecke, E Lalli, and M C Zennaro. Aldosterone-producing adenoma formation in the adrenal cortex involves expression of stem/progenitor cell markers. *Endocrinology*, en.2011-1205, Oct 2011.

5. Sheerazed Boulkroun, Benoit Samson-Couterie, José-Felipe Golib Dzib, Hervé Lefebvre, Estelle Louiset, Laurence Amar, Pierre-François Plouin, Enzo Lalli, Xavier Jeunemaitre, Arndt Benecke, Tchao Meatchi, and Maria-Christina Zennaro. Adrenal cortex remodeling and functional zona glomerulosa hyperplasia in primary aldosteronism. *Hypertension*, 56(5):885–92, 2010.

6. Guillaume Brysbaert, Franois-Xavier Pellay, Sebastian Noth, and Arndt Benecke. Quality assessment of transcriptome data using intrinsic statistical properties. *Genomics, Proteomics, & Bioinformatics*, 8(1):57–71, January 2010.

7. Caroline Colijn, Aaron Brandes, Jeremy Zucker, Desmond S Lun, Brian Weiner, Maha R Farhat, Tan-Yun Cheng, D Branch Moody, Megan Murray, and James E Galagan. Interpreting expression data with metabolic flux models: predicting Mycobacterium tuberculosis mycolic acid production. *PLoS Comput Biol*, 5(8):e1000489, 2009.

8. Sebastian Eilebrecht, François-Xavier Pellay, Peter Odenwälder, Guillaume Brysbaert, Bernd-Joachim Benecke, and Arndt Benecke. EBER2 RNA-induced transcriptome changes identify cel-

lular processes likely targeted during Epstein Barr Virus infection. *BMC research notes*, 1:100, 2008.

9. Paul A Jensen and Jason A Papin. Functional integration of a metabolic network model and expression data without arbitrary thresholding. *Bioinformatics*, 27(4):541–547, 2011.

10. Livnat Jerby, Tomer Shlomi, and Eytan Ruppin. Computational reconstruction of tissue-specific metabolic models: application to human liver metabolism. *Molecular Systems Biology*, 6:401, 2010.

11. E Letavernier, S Peyrard, L Amar, F Zinzindohoue, B Fiquet, and P F Plouin. Blood pressure outcome of adrenalectomy in patients with primary hyperaldosteronism with or without unilateral adenoma. *J Hypertens.*, 26:1816–23, 2008.

12. Sebastian Noth, Guillaume Brysbaert, François-Xavier Pellay, and Arndt Benecke. High-sensitivity transcriptome data structure and implications for analysis and biologic interpretation. *Genomics, Proteomics, & Bioinformatics*, 4:212–29, 2006.

13. Jan Schellenberger, Junyoung O Park, Tom M Conrad, and Bernhard Ø Palsson. BiGG: a Biochemical Genetic and Genomic knowledgebase of large scale metabolic reconstructions. *BMC Bioinformatics*, 11(1):213, 2010.

14. T Shlomi, M Cabili, M Herrgård, Bernhard Ø Palsson, and E Ruppin. Network-based prediction of human tissue-specific metabolism. *Nat Biotechnol*, 2008.

15. Martin I Sigurdsson, Neema Jamshidi, Jon J Jonsson, and Bernhard O Palsson. Genome-scale network analysis of imprinted human metabolic genes. *Epigenetics : official journal of the DNA Methylation Society*, 4(1):43–6, 2009.
