# Supplementary figures and images for: A network perspective on metabolic inconsistency
Source: BMC Syst Biol. 2012 May 14;6:41. doi: 10.1186/1752-0509-6-41 (PMC3579709; doi:10.1186/1752-0509-6-41)

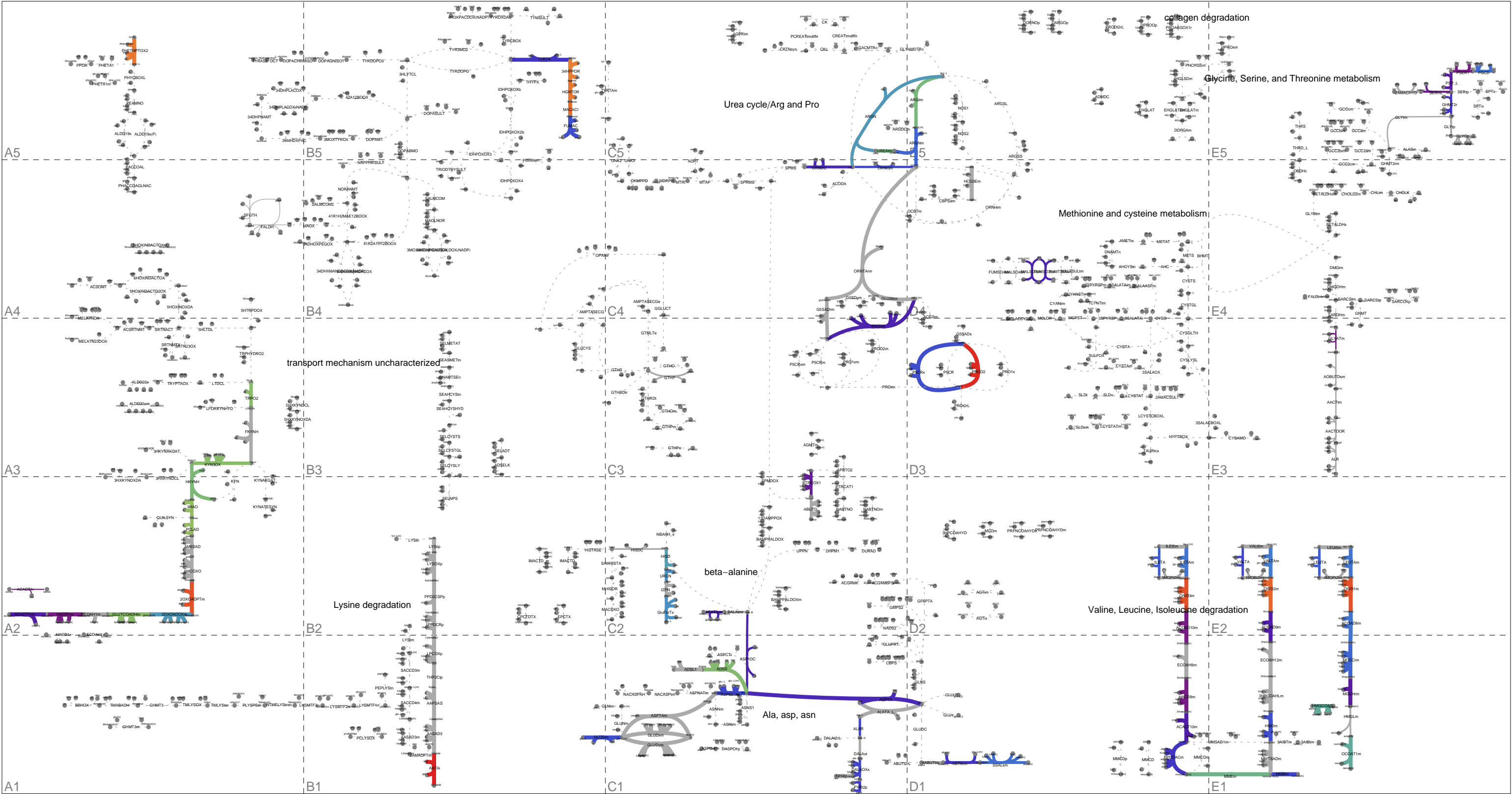

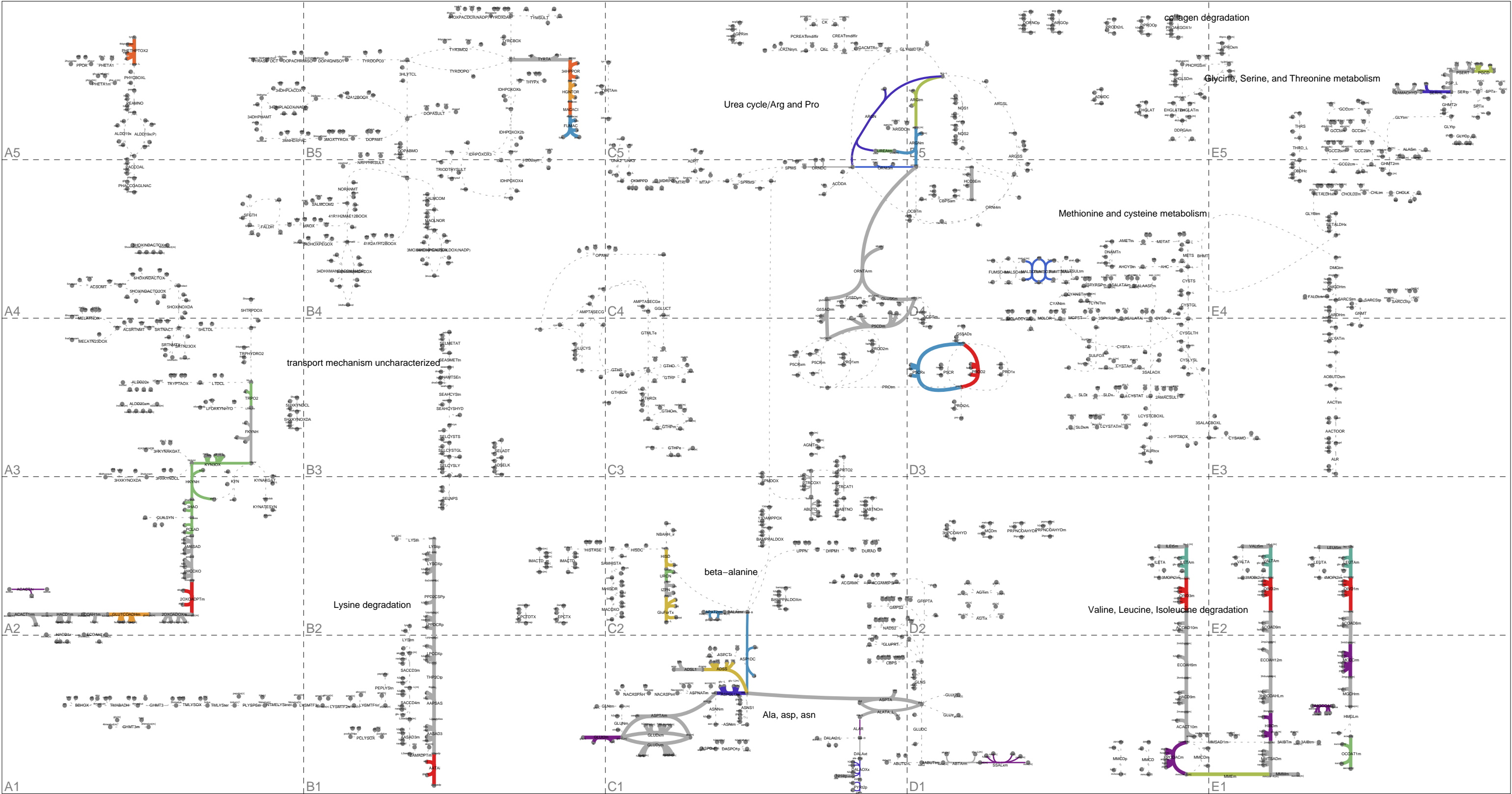

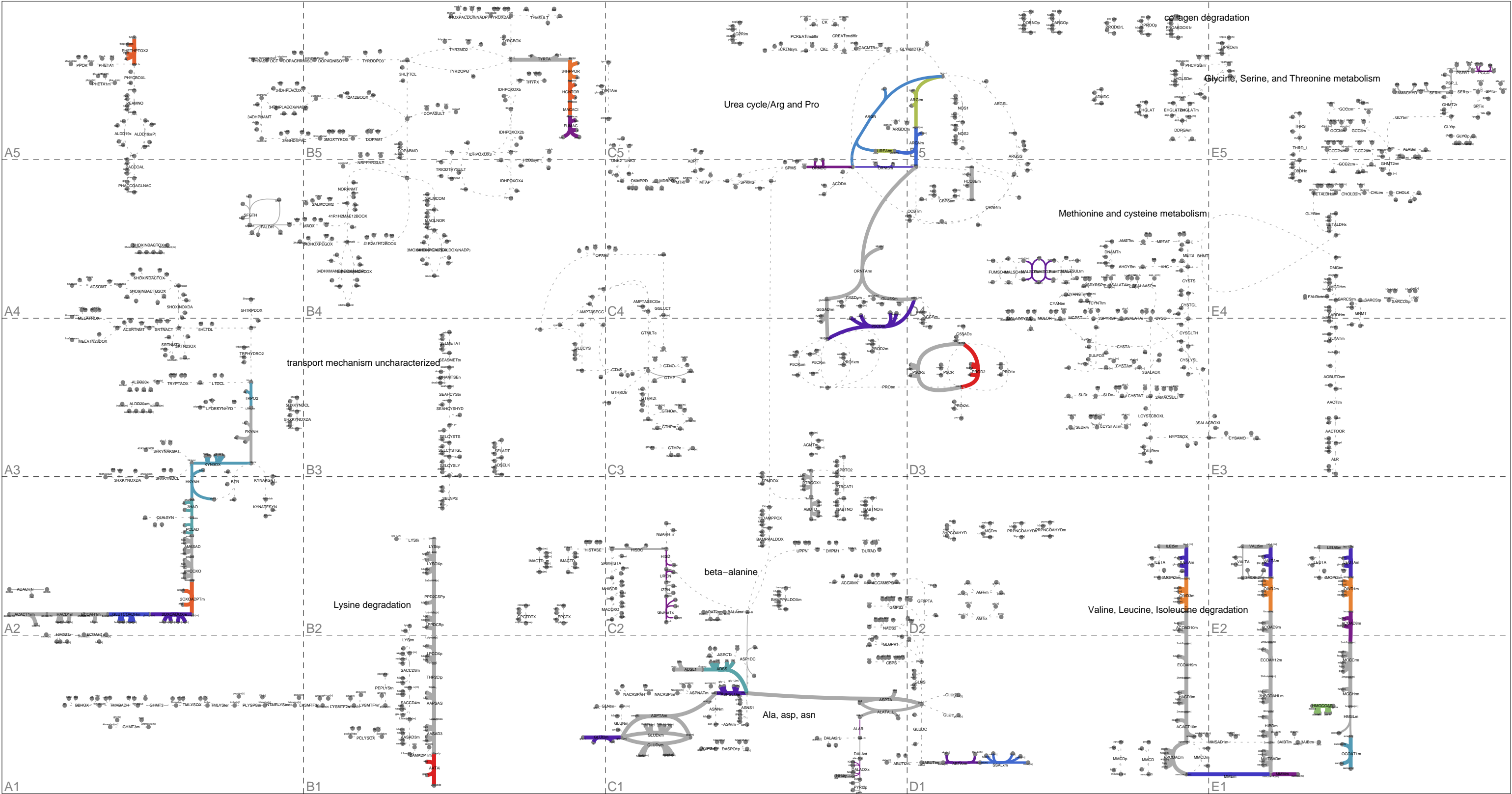

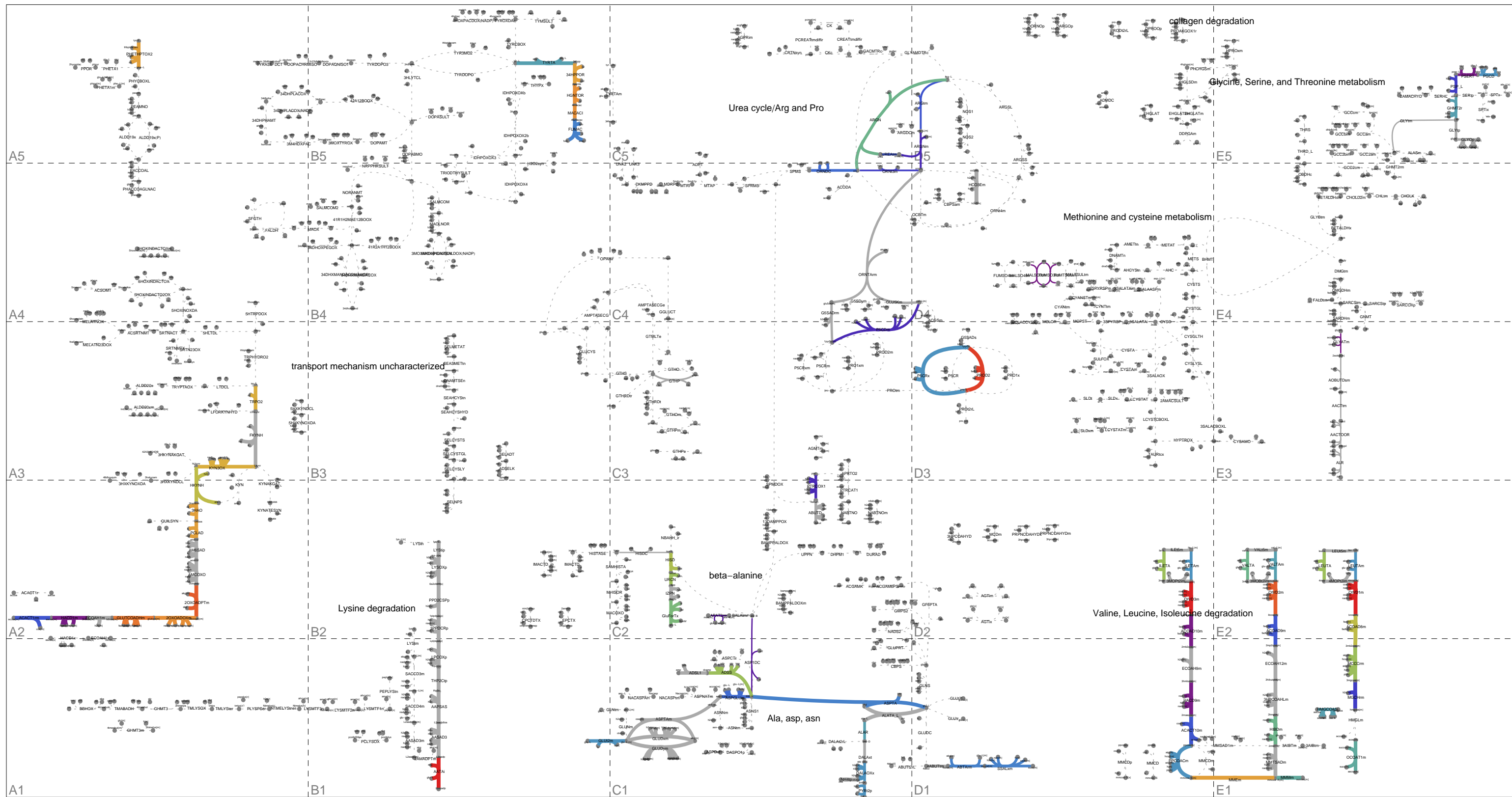

Supplement: Additional file 2 — Pathway map SIaa. Amino acid biosynthesis pathways. The map depicts the usage patterns and inconsistency contributions for the overall contributions (page i), control (page ii), LIG (page iii), and HIG (page iv). The thickness and color of a reaction edge corresponds to the usage frequency and the contribution strength, respectively. The pathway maps have been obtained from the BIGG database [17]. [file 1752-0509-6-41-S2.pdf]

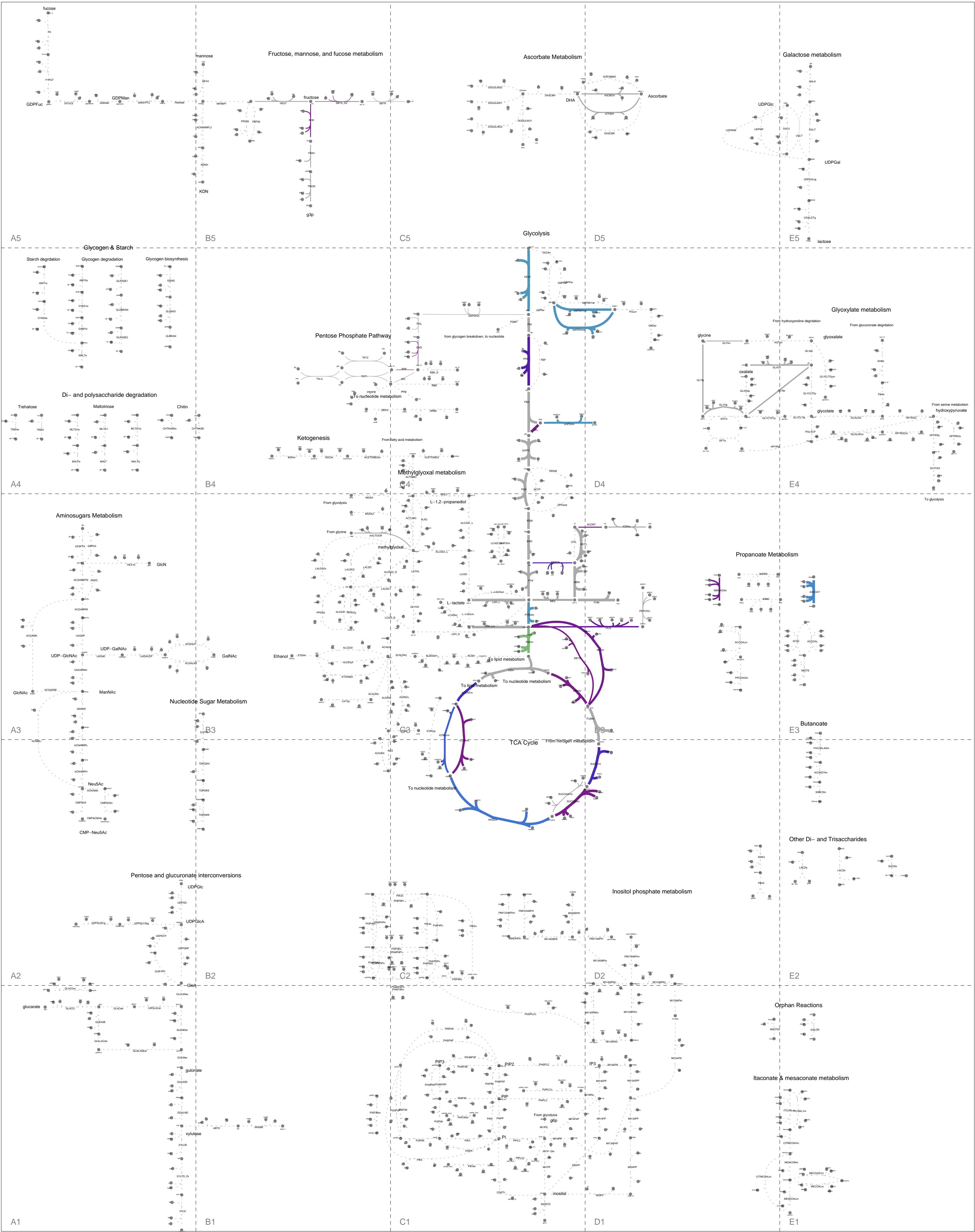

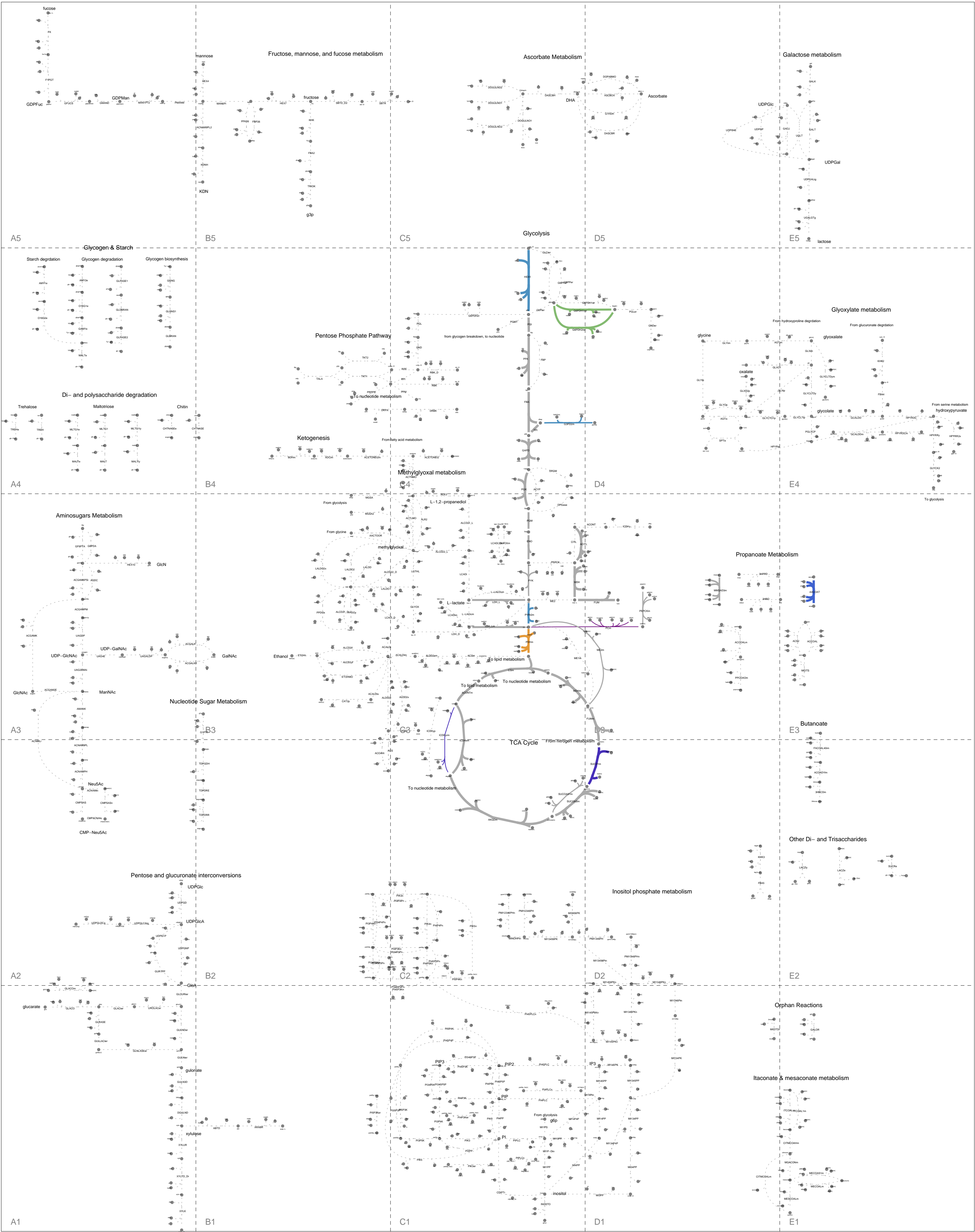

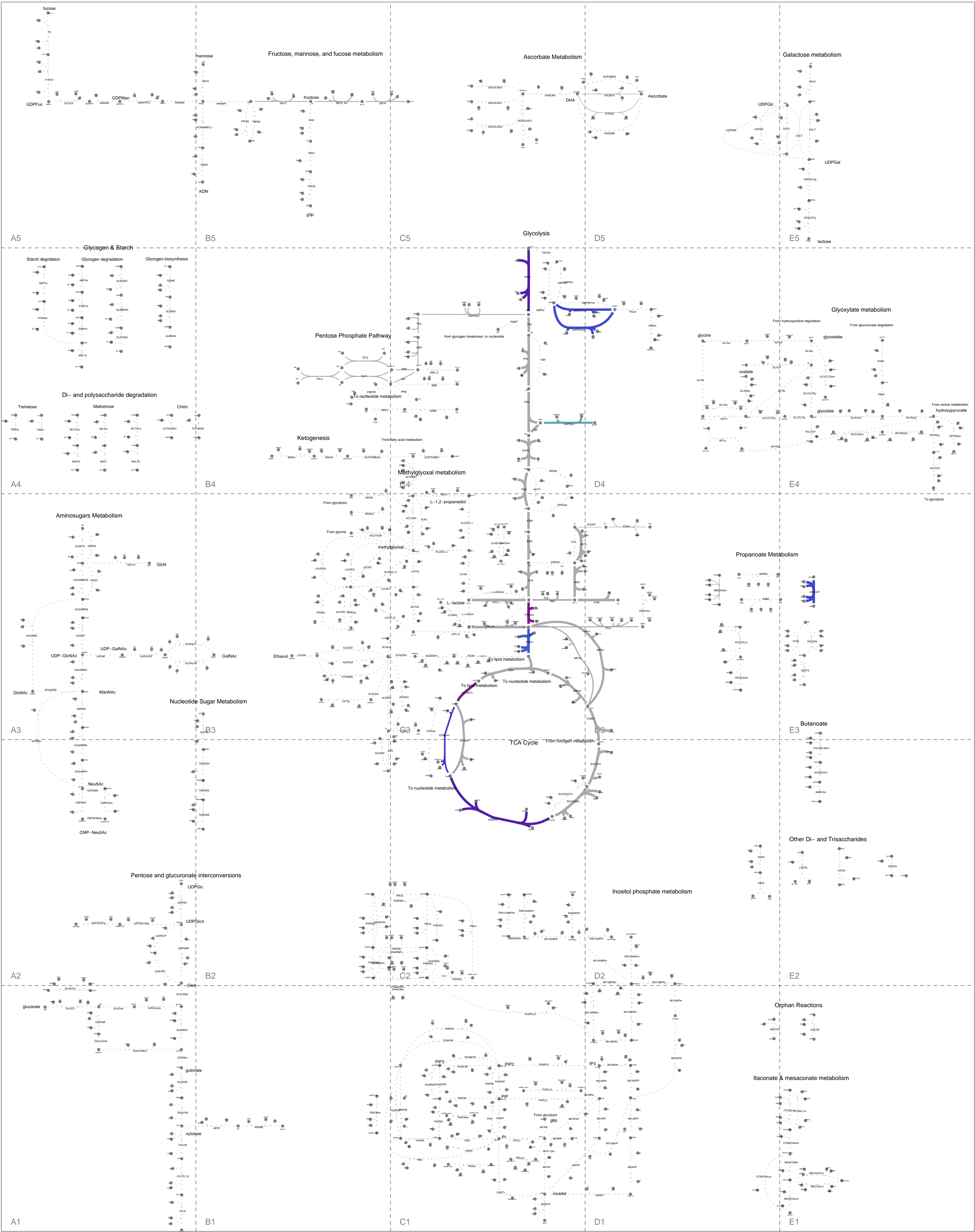

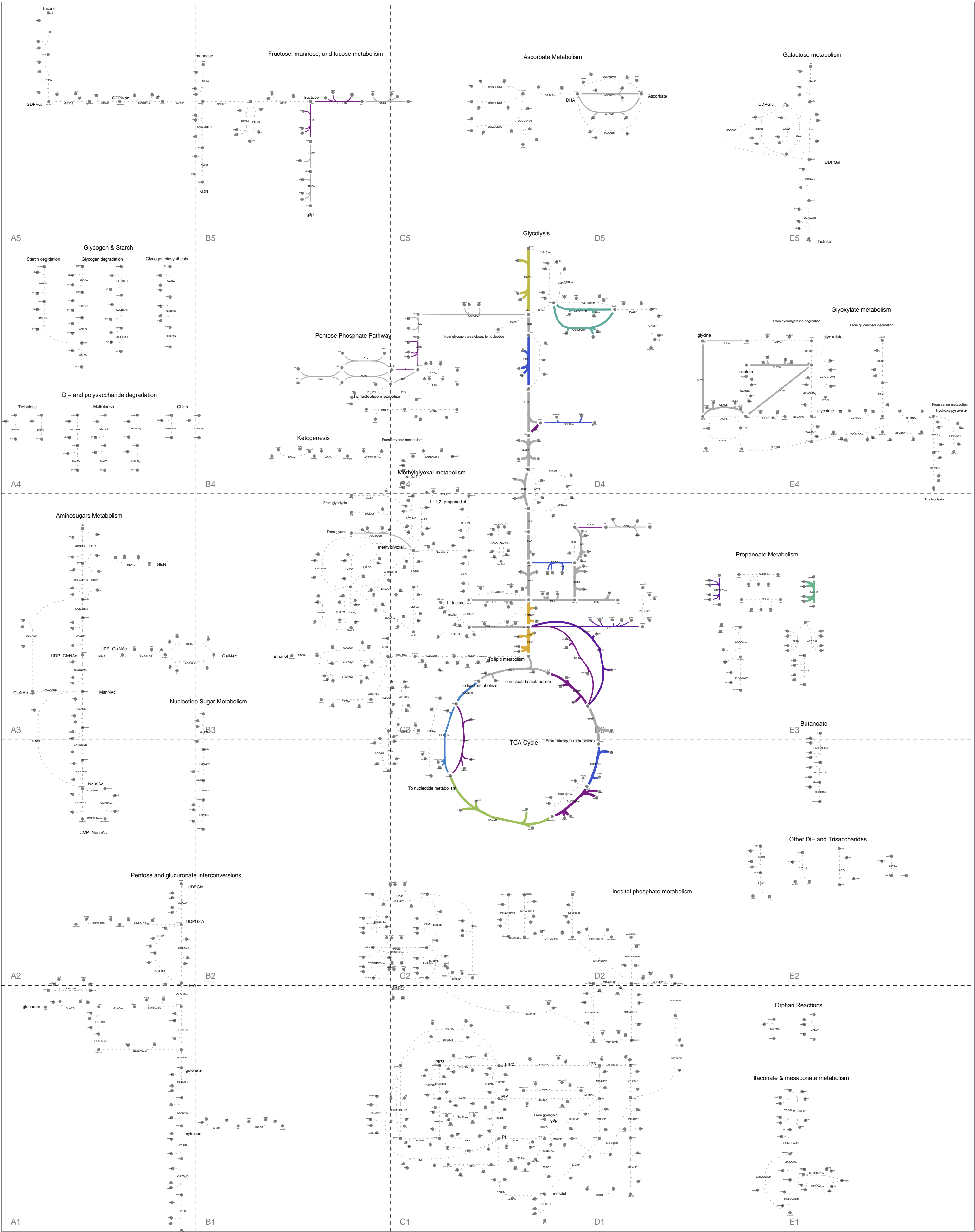

To glucuronate degradation

Supplement: Additional file 3 — Pathway map SIcarb. Central metabolism and carbohydrate pathways (see also Additional file 2. [file 1752-0509-6-41-S3.pdf]

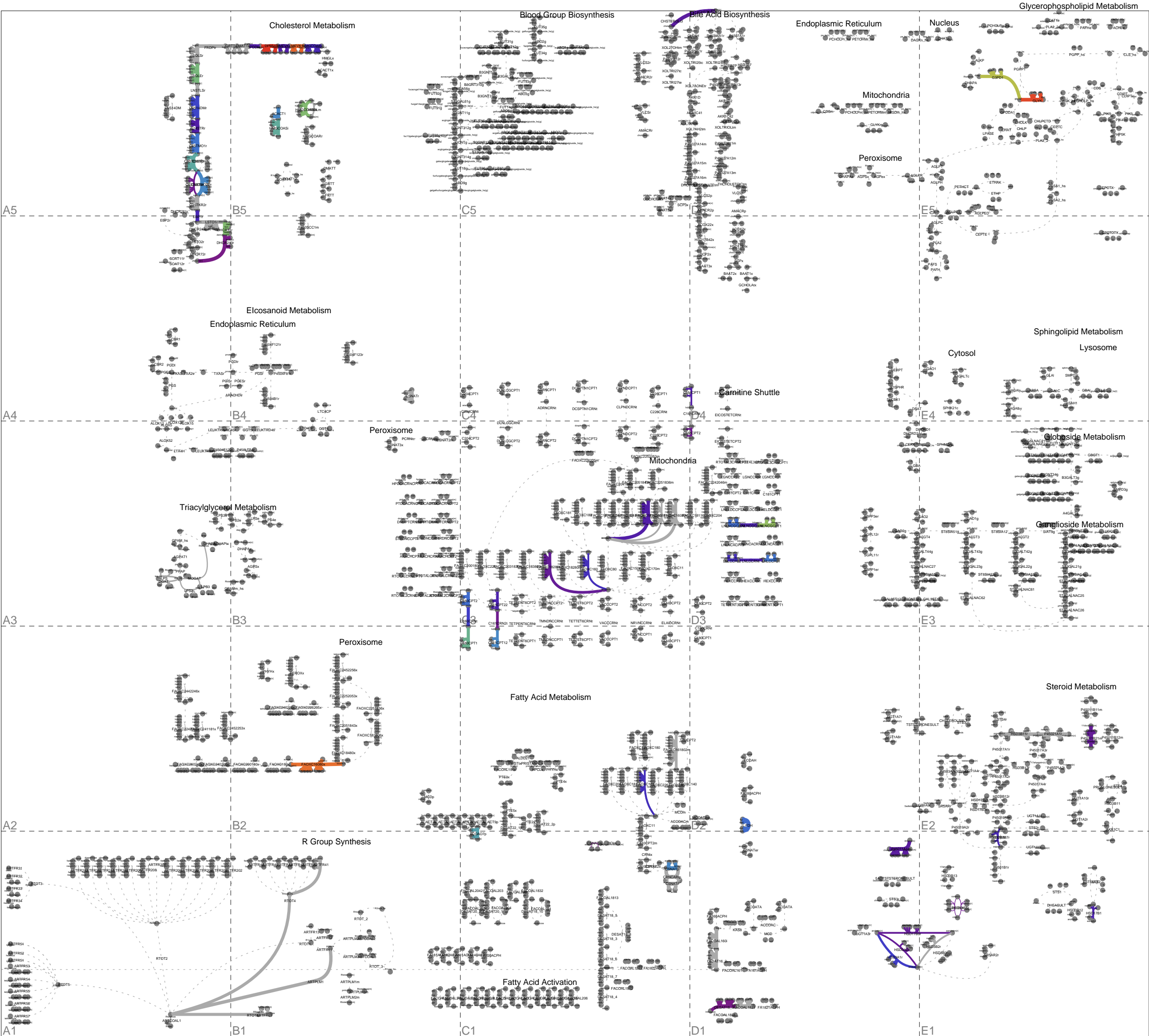

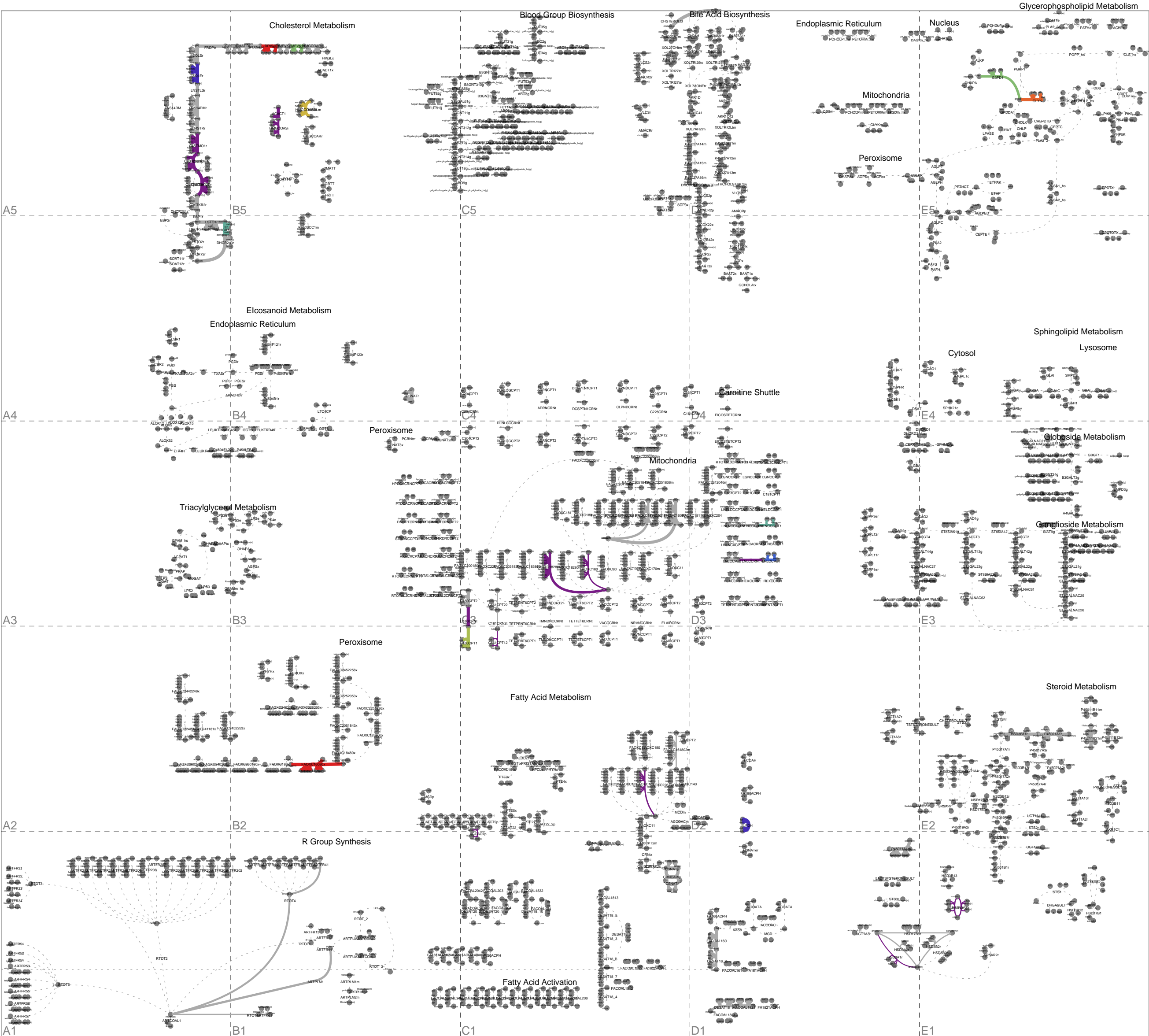

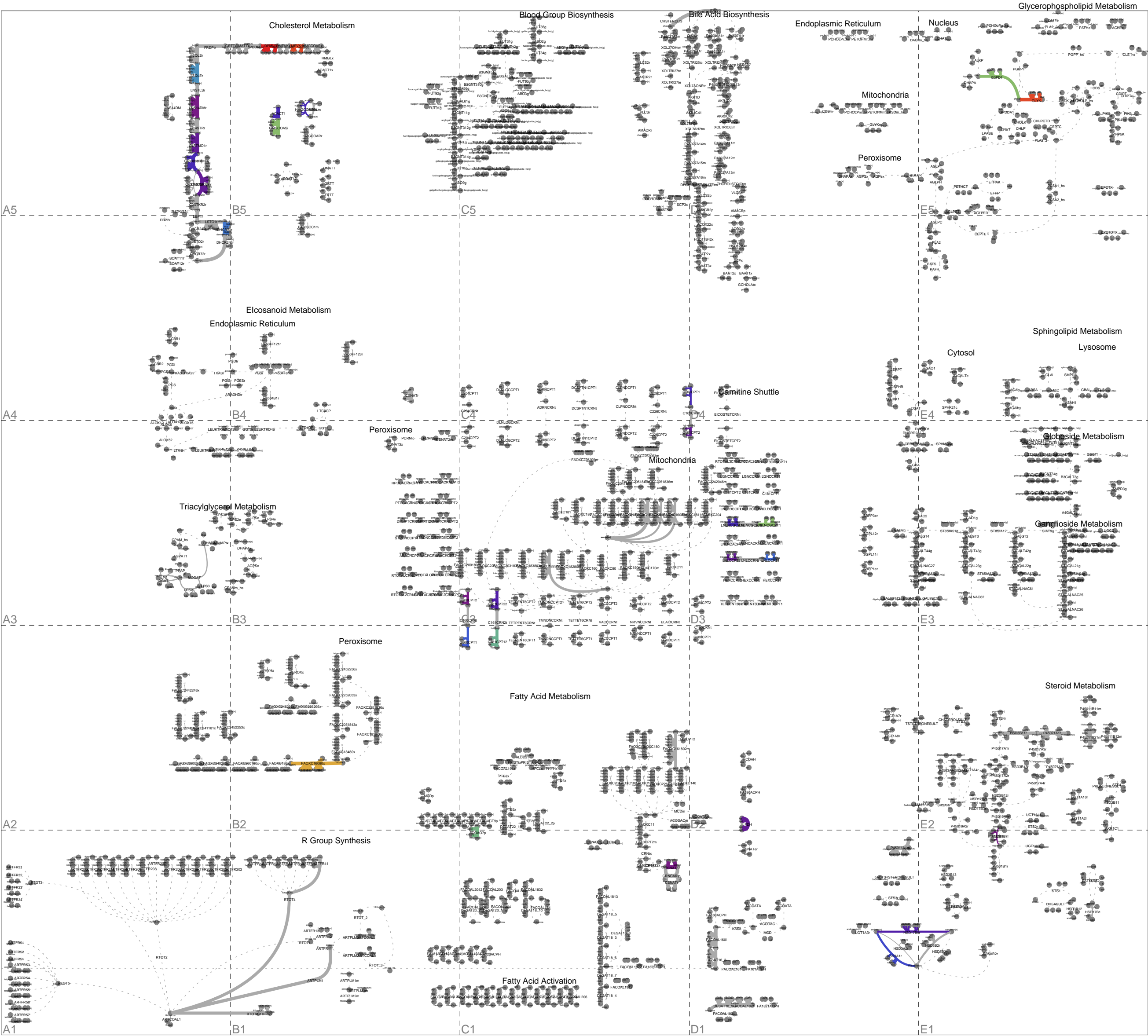

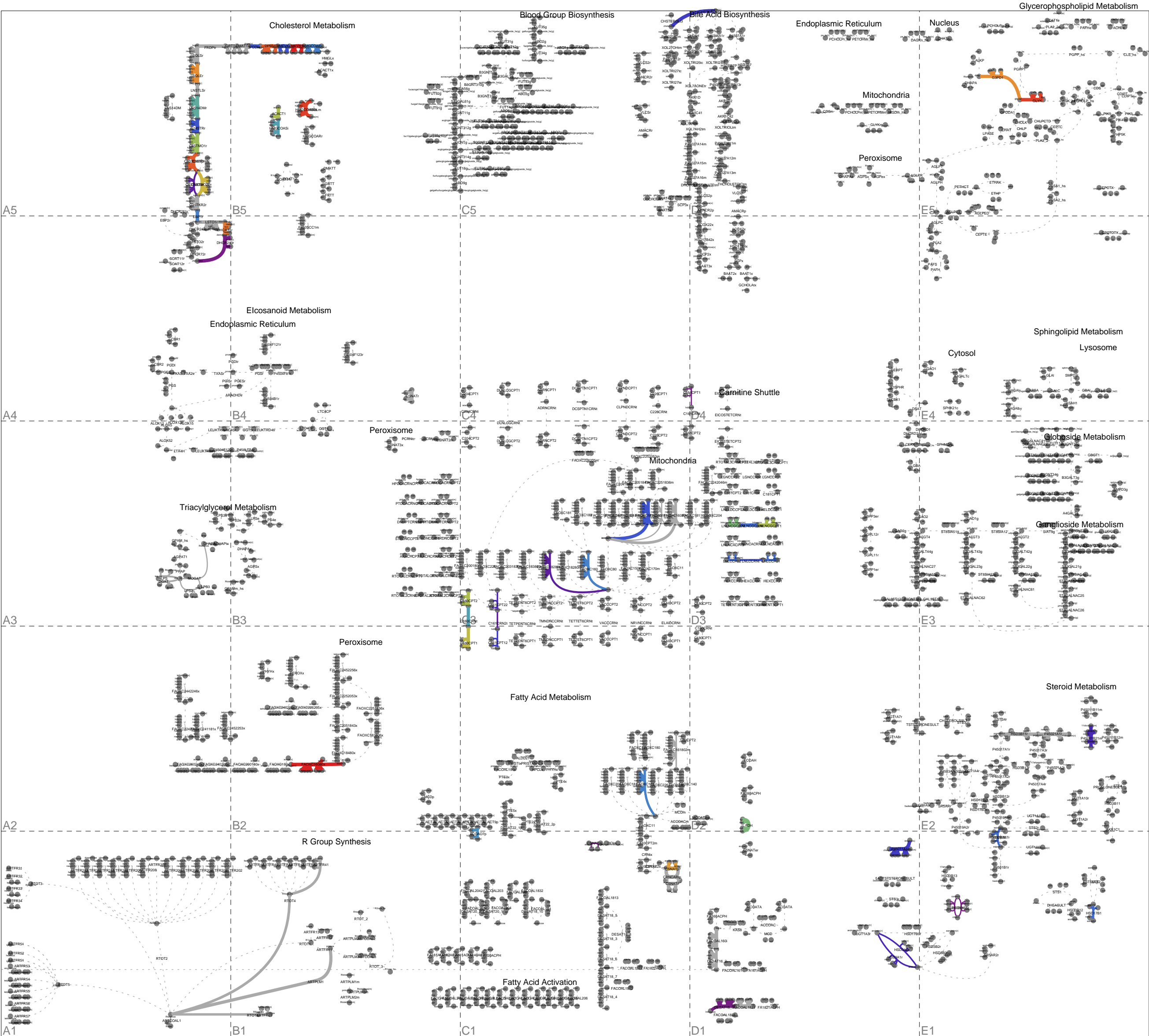

Supplement: Additional file 4 — Pathway map SIlip. Lipid metabolism pathways (see also Additional file 2. [file 1752-0509-6-41-S4.pdf]

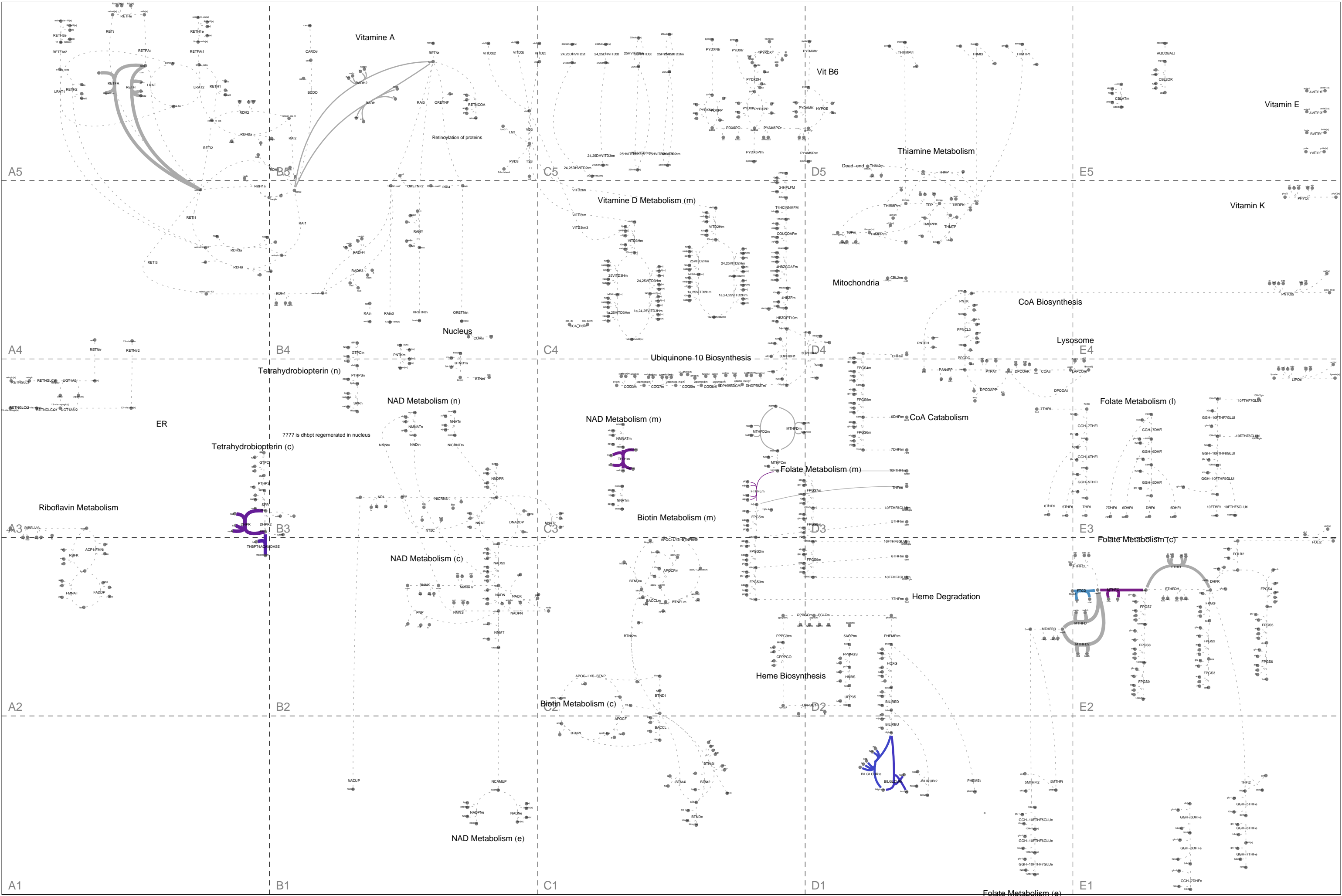

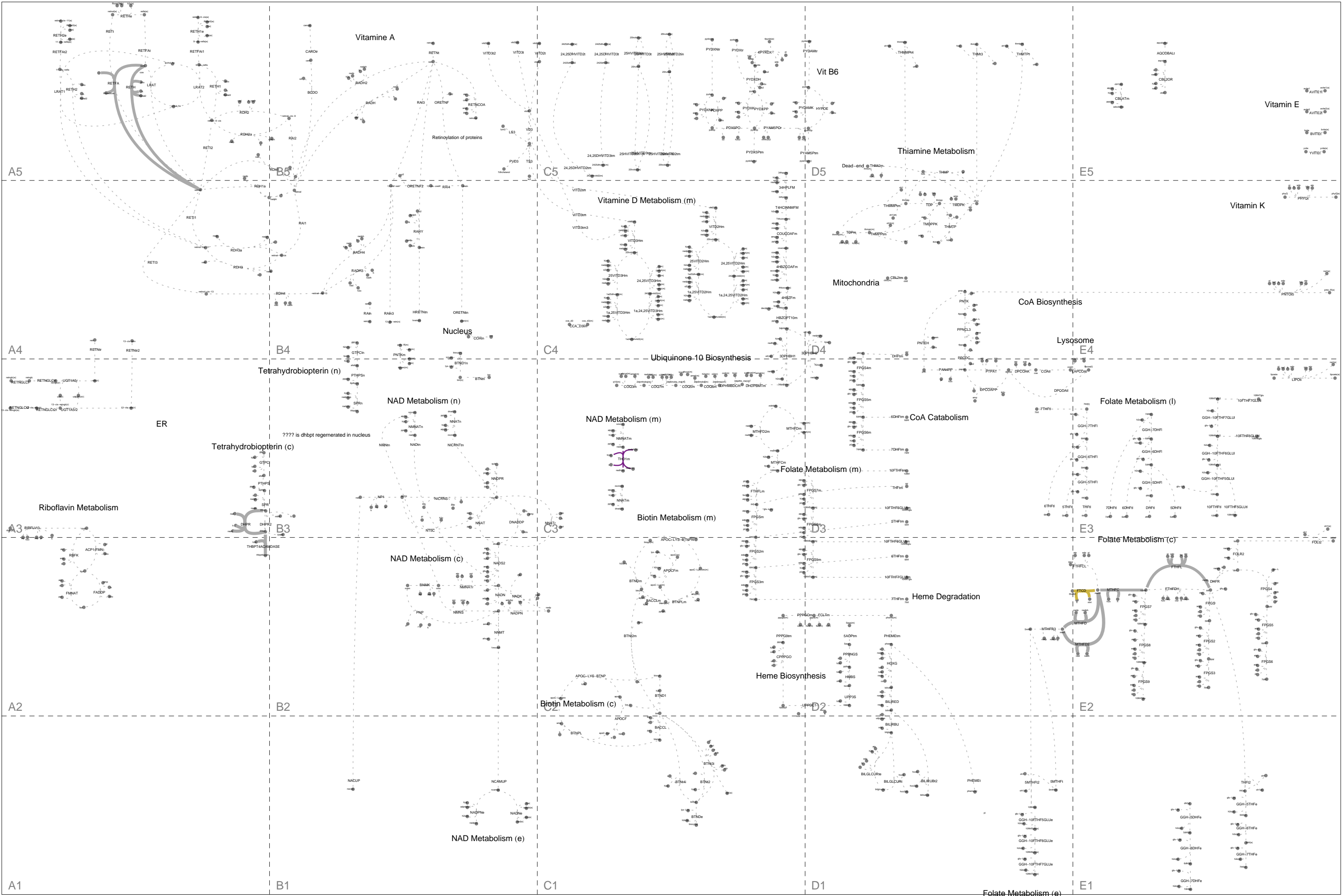

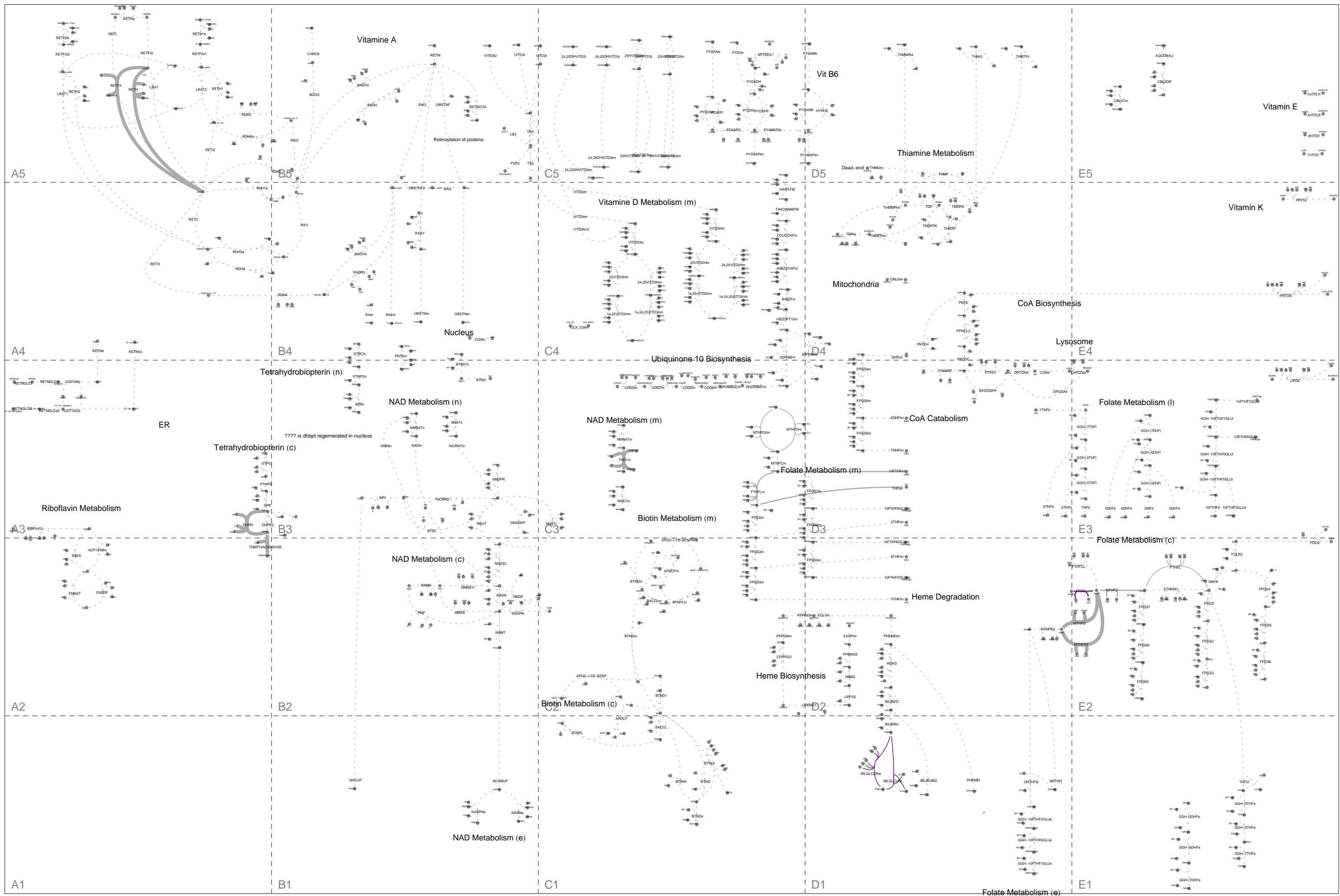

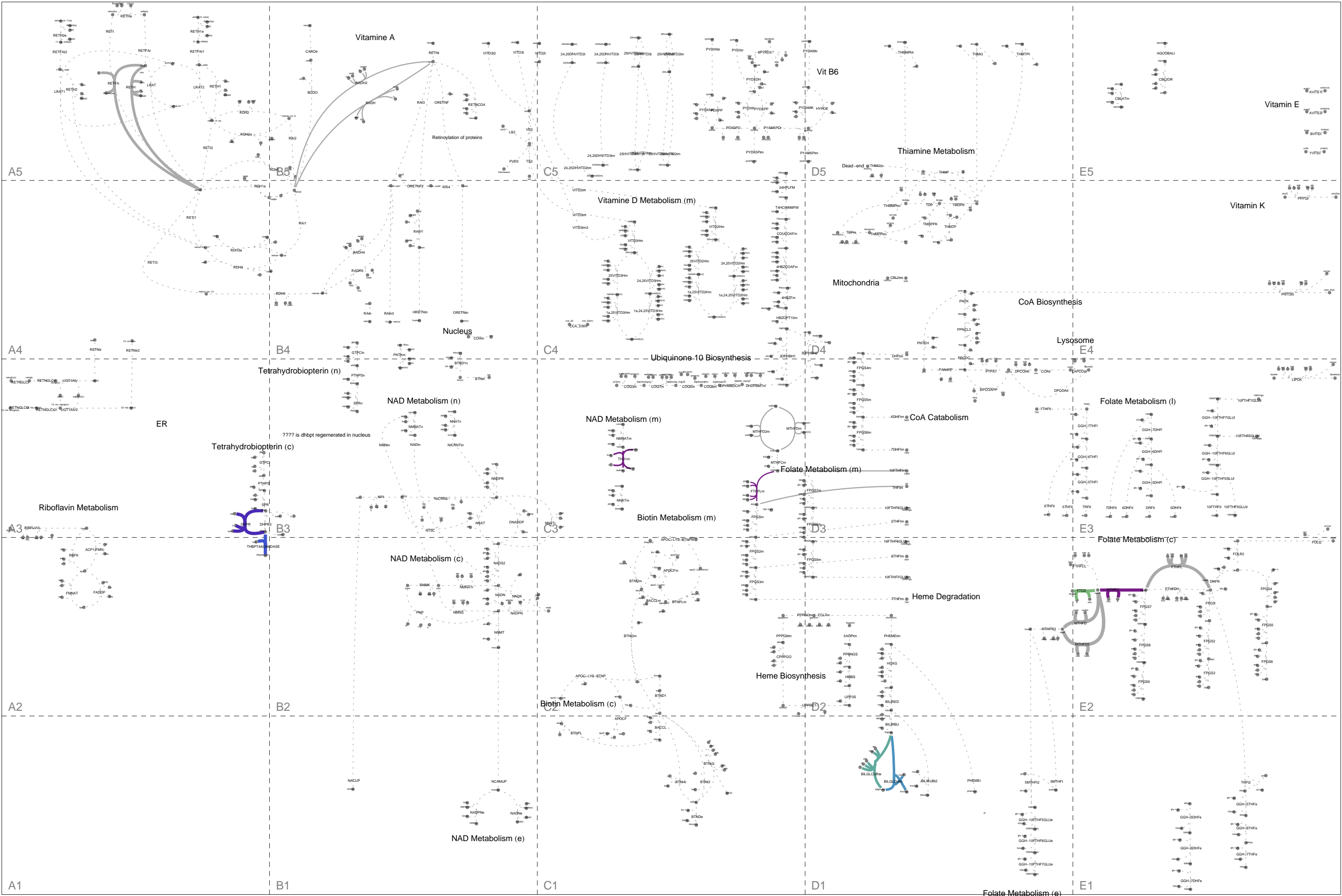

Supplement: Additional file 5 — Pathway map SIvit. Vitamins and cofactor pathways (see also Additional file 2. [file 1752-0509-6-41-S5.pdf]
